# Western Flora

**Published:** 1834

**Authors:** John L. Riddell


### The Western Journal

OF THE

### MEDICAL AND PHYSICAL SCIENCES.

OCTOBER, NOVEMBER, DECEMBER, 1834.

### ESSAYS AND CASES.

### Art. I.—Western Flora.

*Synopsis of the Flora of the Western States.*—By John L. Riddell.

PREFATORY REMARKS.

The region to which this synopsis or catalogue is intended to apply, extends from the Alleghany mountains in West Virginia, to the Platte river in Missouri Territory; and from the southern boundary line of Tennessee, to the latitude of Detroit. It embraces Ohio, Indiana, Illinois, Kentucky, West Tennessee and Missouri; a small part of Virginia and Pennsylvania, and of the Michigan, North West, and Missouri territories. There are large tracts of country within these limits, whose vegetable productions have not yet been examined by botanists;—even in the immediate neighborhood of populous towns and cities, much remains to be done. Judging from available information, it would seem that the botany of Ohio and Kentucky has been most satisfactorily determined. The Kentucky plants are given almost exclusively in the authority of Dr. Short; those of Ohio, unless otherwise accredited, have been personally observed and collected by the writer. The regions about St. Louis, on either side of the Mississippi, have been very successfully explored by Dr. L.

C. Beck, whose communications on the subject may be found in Silliman's Journal.

We are chiefly indebted to the eminent naturalist, Mr. Nuttall, for our knowledge of the botany of Missouri Territory: though the researches of Mr. Bradbury and Dr. James have aided very materially. The author regrets that he has been unable to obtain definite and satisfactory knowledge in regard to the productions of Tennessee. Perhaps enough has been gleaned, however, from the writings of Pursh and Nuttall, to apologise for not excluding that State.

It has for several years been the author's design, to publish a Flora of the Western States, when he shall have accumulated a sufficiency of materials; and he takes this opportunity of soliciting information from those who may choose to favour him with their correspondence, and of proposing an interchange of botanical specimens, with all who may wish to form collections. The following catalogue, though necessarily incomplete, will probably aid in effecting the desired object, by exhibiting its present state of advancement, thereby enabling observers located in different sections of the assumed territory, the more easily to make additions to it.

Among the scientific gentlemen residing in the West, to whom the student in botany can refer with advantage, may be mentioned Dr. C. W. Short, Dr. R. Peter. and Mr. H. J. Griswold, of Lexington, Ky.; Dr. D. Houghton, of Detroit, Mich.; Dr. T. Townsend, Wheeling, Va.; Mr. J. R. Paddock, Worthington, Ohio; Mr. J. W. Van Cleve, Dayton, Ohio; Dr. J. Eberle, Dr. J. Locke, Dr. Colby, Dr. Frank, and Mr. R. Buchanan, of this city.

The nomenclature here adopted, is in general accordance with the sixth edition of Eaton's manual. This observation is made in order to dispense as far as possible with the citation of authority. The philosophic method of Prof. Lindley is observed in the arrangement:—the reader is doubtless aware that the natural system of botany, as it is called, aims to aggroup plants according to their affinities, or general resemblances.

Botanists consider the vegetable world as made up of two great divisions, which have been denominated *Vasculares* and *Cellulares*. They differ from each other in their intimate organization and in their modes of reproduction. The *vascular* division embracing all the true flowering plants, is characterized by the presence of minute tubes, formed by the coiling of the vegetable fibre in a spiral manner, and hence called spiral vessels. The *Cellulares* are destitute of spiral vessels.

The *Vasculares* are constituted by two great and well marked classes;—the *exogenæ* and the *endogenæ*. The *exogenæ* if perennial, have their vessels and cellules disposed in concentric cylinders; they possess bark and pith, and increase by annual depositions of new matter between the bark and wood, as well as by the protrusion and development of buds; as the oak, the rose, the milkweed. *Endogenous* plants have neither pith nor bark. Their vessels are irregularly disposed in fasciculi and surrounded by cellular tissue. While the trunk or stem remains soft and distensible, it may enlarge by the formation of new substance interiorly; but after it becomes indurated, the magnitude of the plant is increased solely by the development of the terminal bud or buds; as is exemplified in the palms, canes and grasses.

The grand division *Cellulares*, corresponds pretty closely with the *Cryptogamia* of Linnæus. It comprises the flowerless plants, which are alike destitute of spiral vessels and of the true vegetable fibre. Some of them, as the simpler fungi, appear to consist wholly of membranous vesicles, which like soap bubbles in a phial, by their contiguity and mutual pressure become hexagonal cells. The mosses, lichens, algæ, and the more highly developed fungi, though seemly more complex in their structure, have nothing it is asserted but the cellular tissue in their composition. Besides innumerable contiguous cellules which are common to all vegetables, the ferns, horsetails and ground pines possess membranous tubes, very similar to the reducent vessels which traverse the inner bark of trees.

### Characters and Abbreviations Explained.

*Time of flowering.*

Ap. April.
M. May.
Ju. June.
Jul. July.
Aug. August.
Sept. September.
Oct. October.

*Colour of the Flower.*

*r*. Red. *p*. Purple. *y*. Yellow.
*w*. White. *b*. Blue. *g*. Green.

*Height of the Plant.*

1 f. One foot. 2 i. Two inches.

*Duration, &c.*

⊙ Annual. ♂ Biennial. ♃ Perennial. ♄ Woody.

*Particular Situations.*

dp. Dry prairies.
wp. Wet prairies.
mh. Marshes.
pl. Standing pools.
rb. Denuded river banks.
bt. Bottom lands.
md. Meadows.
cf. Cultivated fields.
rd. Road sides.
rs. Rocky situation.
sr. Slaty ravines.
cr. Calcareous ravines.
ow. Open woods.
sw. Dense and shady woods.
br. The bed of rivulets.
bg. Shaking bogs.

*Localities.*

O. Ohio.
Ky. Kentucky.
Ia. Indiana.
Ill. Illinois.
Mich. Michigan.
Tenn. Tennessee.
Ark. Arkansas.
Mo. Missouri.
Mo. Ter. Missouri Ter.
W. Western States.
Mar. Marietta, O.
Col. Columbus, O.
Wor. Worthington, O.
Cin. Cincinnati, O.
Mi. Miami country, O.
Plt. Platte river, Mo. Ter.
C. Bl. Council Bluffs.
St. L. St. Louis.
Miss. Mississippi river.
Intr. Introduced.

*By whom observed.*

Nutt. Nuttall. Bk. Beck. St. Short. Jas. James.

### VASCULARES.

### *CLASS I.—EXOGENÆ.*

TRIBE I.—ANGIOSPERMÆ.—*Seeds in some kind of covering.*

DIVISION I.—Flowers with many petals or none.

ORDER I. *ARALIACEÆ.*—THE GENSING TRIBE.

1. Aralia spinosa. Angelica tree. Shot bush. Aug. *w.* 10 f. ♄. sw. Ky. *St.* Miss. *Bk.* Stimulant, diaphoretic, emetic and cathartic if recent.
2. A. racemosa. Spikenard. Aug. *w.* 4 f. ♃. ow. W. Healing, pectoral, stimulant and diaphoretic.
3. A. nudicaulis. False sarsaparilla. Jul. *g.* 1½ f. ♃. ow. Mar. O. Stimulant diaphoretic, alterative. Raf. Med. Flor. i. 33.
4. Panax quinquefolia. Ginseng. Ju. *g.* 1 f. ♃. ow. W. Warming and invigorating stomachic.
5. P. trifolia. Dwarf ground nut. May. *w.* 4 i. ♃. ow. War. O. Root spherical, nutritious.

II. *UMBELLIFERÆ.*—THE UMBELLIFEROUS TRIBE.

6. Erigenia bulbosa, *Nutt.* Turkey pea. March–Ap. *r–w.* 4 i. ♃. ow. W. Root similar to No. 5.
7. Sanicula marilandica. Indian sanicle, often called black snakeroot. Jul. *g–w.* 2 f. ♃. ow. W. Diaphoretic, stimulant, stomachic.
8. Eryngium aquaticum. Button snakeroot. Aug. *w–b.* 3 f. ♃. wp. Darby Plains, O. Diaphoretic, expectorant.
9. E. ovalifolium. Sea holly. Jul. *b.* 2 f. ♃. Near the Yellow Springs. *N. Longworth*, Esq.
10. Cicuta maculata. Water hemlock. Jul.–Aug. *w.* 4 f. mh. md. O. Ky. Narcotic. Big. Med. Bot. i. 125.
11. C. bulbifera. Bulb-bearing water hemlock. Aug. *w.* 3 f. ♃. mh. O.
12. Sium latifolium. Water parsnip. Jul. *w.* 3 f. mh. W.
13. S. tricuspidatum. Jul. 3 f. mh. St. L. *Bk.*
14. Archemora rigida, *De Cand.* Sept. *w.* 3 f. mh. wp. O.
15. A. ambigua, *De Cand.* Aug. *w.* 4 f. ♃. Sandy Swamps. Mich. *Gray.*
16. Zizia cordata. Alexanders. May. *y.* 2 f. ♃. sr. O.
17. Z. aurea. Meadow parsnip. June. *y.* 1 to 2 f. ♃. sr. O.
18. Z. integerrima. June. *y.* 1 to 2 f. ♃. rs. W.
19. Thapsia trifoliata. *y.* 3 f. bt. St. L. *Bk.*

20. Ligusticum actæifolium. Jul. ♃. dp. 3 f. O.
21. Angelica triquinnata. Archangel. Aug. *w.* 3 f. ♃. dp. O. Aromatic, tonic.
22. A. atropurpurea. Masterwort. June. *w.* 5 f. ♃. bt. Wor. O. Stimulant, conservative, antilithic.
23. A. lucida. Jul. *w.* 2 f. ♃. dp. (sw. *Bk.*) O. Aromatic root.
24. Heracleum lanatum. Cow parsnip. Masterwort. June. *w.* 5 f. md. Ky. *St.* Stimulant, carminiative. Wood and Bache, Disp. 335.
25. H. sphondylium. ♂. Mo. Ter. *Nutt.* Grand riv. *Jas.*
26. Ferula fœniculacea. May. *y.* 1 f. ♃. Grand riv. *Jas.*
27. F. villosa. Jul. *w.* ♃. Louisville. *M'Murtrie.*
28. Pastinaca sativa. Parsnip. Jul. 3 f. *y.* ♂. cf. Introduced.
29. Uraspermum claytoni. Sweet cicely. June. *w.* 2 f. ♃. sw. W. The root is very fragrant and of a fine flavor, nearly resembling paragoric.
30. U. hirsutum. Hairy cicely. Poison cicely. June. *w.* 1½ f. ♃. sw. W.
31. Myrrhis canadensis, *Nutt*, Canadian Cicely. June. *w.* ♃, ow. O.
32. Chærophyllum procumbens, *Nutt.* Dwarf cicely. May. *w.* 10 i. ♃. bt. ow. O. Root fragrant.
33. Seseli divaricatum. May. *y.* 6i. ♃. rb. Missouri riv. *Nutt.*
34. Conium maculatum. Poison hemlock. Cicuta. Jul. *w.* 4 f. ♃. rd. W. Probably introduced. Narcotic. Big. Med. Bot. i. 113. Raf. Med. Fl. i. 118.
35. Daucus carota. Wild carrot. Jul. *w.* 2 f. ♃. md. Old fields. Hardly yet naturalized. Excitant, diuretic.

### III. *RANUNCULACEÆ.*—The crow foot tribe.

#### I. Clematideæ.—*Virgin bower like.*

36. Clematis virginica. Virgin's bower. Aug. *w.* Vine. ♃. ow. rs. W.

37. C. viorna. Leather flower. Jul. Flowers violet. Vine. ♃. ow. md. Wor. Mi. O.

38. C. reticulata. Jul. *p–r.* ♃. Vine. Ky. *St.* St. L. *Bk.*

#### II. Anemoneæ.—*Windflower like.*

39. Anemone virginiana. Wind flower. Jul. *w.* 1½ f. ♃. md. ow. W.

40. A. aconitifolia. Wolf bane wind flower. Jul. *w.* 1 f. ♃. md. W.

41. A. nemorosa. Low anemone. Ap. 6 i. *w.* ♃. ow. W.

42. A. ludoviciana. Ap. *r–b.* ♃. Mo. Ter. *Nutt. Jas.*

43. A. tenella, *Pursh.* March. *w.* ♃. Plt. *Jas.*

44. Thalictrum dioicum. Meadow rue. Ap. *w.* 2 f. ♃. md. sr. W.

45. T. corynellum. Jul. *w.* 4 f. ♃. W.

46. T. ranunculinum. Ky. *St.*

47. T. cornuti. Prairie rue. Jul. *g–y.* 4 f. ♃. wp. O.

48. T. rugosum. Swamp rue. Aug. *w.* 5 f. ♃. Swamps. West Va.

49. T. revolutum. Aug. *w.* 2½ to 3 f. ♃. Mar. O.

50. T. anemonoides, *Michx.* Rue leaved anemone. Ap. *w.* 6 i. ♃. ow. W.

51. Hydrastis canadensis. Yellow puccoon. Ohio curcuma. Yellow root. May. *r–w.* 8 to 10 i. ♃. sw. W. Stimulant, tonic, detergent. Bart. Med. Bot. ii. 17.

52. Hepatica acutiloba. Liver-leaf. Ap. *w–p.* 3 i. ♃. sw. W. Medicinal. See No. 53.

53. H. americana. Blunt lobed liver-leaf. Ap. *w.* 3 i. ♃. sw. Wor. O. Demulcent, astringent, diuretic, deobstruent.

III. Ranunculæ.—*Crow foot like.*

54. Ranunculus flammula. Small spearwort. Jul.–Sept. *y.* 1½ f. ♃. mh. W. Acrid, rubefacient, vesicatory.
55. R. pennsylvanicus. Jul. *y.* 1½ f. ♃. Wet meadows, O.
56. R. abortivus. Ju.–Aug. *y.* 1 f. ♃. br. mh. O. Ky.
57. R. fascicularis. Fascicle rooted butter cup. May. *y.* Woods. W.
58. R. recurvatus. Small flowered butter cup. Ap. Pale *y.* 1 f. ♃. ow. West Va. Cin. Wor. O. Medicinal. See No. 54.
59. R. lanuginosus. Ju. *y.* ♃. Wor. O.
60. R. pusillus. Jul. pale *y.* 10 i. ♃. Wet grounds. Mich. *Houghton.*
61. R. hispidus. Hairy crow foot. Jul. *y.* 18 i. ♃. Wet grounds. W. *Bk.*
62. R. filiformis, *Michx.* Thread form crow foot. Ju. *w–y.* Creeping. ♃. Mich. *Houghton.*
63. R. lacustris, *Bk.* Water crow foot. Lake crow foot. June. *y.* Creeping. 3 to 4 feet long. ♃. mh. pt. Herculaneum. Mo. *Bk.* Darby Plains, O.
64. R. collinus. Ap. 2 i. wp. Fort Clark. *Bk.*
65. R. bulbosus. Butter cups. Jul. *y.* 1 f. ♃. mk. Cin. O. *Eberle.* Louisville. *M'Murtrie.* Medicinal. See species 54. Big. Med. Bot. iii. 61.
66. R. repens. June–Sept. *y.* 1 to 2 f. ♃. Wet md. Louisville. *M'Murtrie.* Medicinal. See Disp. of Wood and Bache, 525.
67. Myosurus Shortii. Mousetail. ⊙. Ky. *Raf.*

IV. Helleborneæ.—*Hellebore like.*

68. Caltha pulastris. Marsh marygold. American cowslip. Cowslops. April. *y.* 8 to 12 i. ♃. mh. O. Herbage boiled for "greens" before flowering.
69. Trollius americanus, *Muhl.* Globe flower. June. *y.* I f. ♃. Wet grounds. W. *Bk.*
70. Coptis trifola. Gold thread. Jul. *w.* 4 to 6 i. ♃. Swamps. Louisville. *M'Murt.* Tonic. Big. Med. Bot. i. 60. Raf. Med. Fl. i. 27.
71. Isopyrum thalictroides, *Linn.* Enemion biternatum, *Raf.* Ap. *w.* 8 i. ♃. ow. Ky. O. Resembles Sp. 50 closely.
72. Aquilegia canadensis. Wild columbine. Ap. *y–r.* 2 f. ♃. rs. Ky. O.
73. Delphinium tricorne. Stagger weed. Three horned larkspur. May. *b.* 1 f. ♃. ow. sw. O.
74. D. azuieum. Azure larkspur. May. *b.* 2 f. ♃. ow.—Near Wheeling.
75. D. exaltatum. Tall larkspur. June. *b.* 3 f. ♃. dp. O. Diuretic, emetic, cathartic. Wood and Bache Disp. 269.
76. D. virescens. *g–w.* 8 to 12 i. ♃. Plains on the Missouri river. *Nutt.*
77. D. consolida. Larkspur. June. *b.* 2 f. O. Introduced. Medicinal. See sp. 75.
78. Aconitum uncinatum. American wolfsbane. Sept. *p.* Twining. ♃. Preston glades. West Va. *Paddock.*

V. Pæonæe.—*Allied to the garden peony.*

79. Macrotys racemosa. Black snakeroot. Black cohosh. Jul. *w.* 5 f. ♃. ow. W. tonic, secernant stimulant, abortivant, mildly astringent.
80. Actæa alba. White cohosh. Necklace weed. May *w.* 18 to 24 i. ♃. sw. W. Medicinal, similar to sp. 79.
81. Xanthorhiza apiifolia. Yellow root. May. *p.* 2 to 3 f. ♄. Louisville. *M'Murt.* Ohio banks. *Nutt.* Tonic bitter. Bart. Med. Bot. ii. 203.

VI. *PAPAVERACEÆ.*—The poppy tribe.

82. Chelidonium majus. Celandine. May–Oct. *y.* 15 i. rd. Near Johnstown, O. Introduced. Acrid, stimulant, detergent, diuretic.
83. Meconopsis diphylla. Western celandine. May. *y.* 1 f. ♃. Woods. W. Closely resembles sp. 82, for which it may doubtless be substituted.
84. M. petiolata. June. *y.* ♃. Lead mines, Mo. *Jas.*

85. Sanguinaria canadensis. Blood root. Puccoon. Ap. *w.* 4 i. ♃. bt. cr. W. Emetic, stimulant, narcotic. Big. Med. Bot. i. 75. Bart. Med. Bot. i. 31.
86. Argemone mexicana. Prickly poppy. Jul. *y.* or *w.* 2 to 3 f. ⊙. Banks of streams. Plt. *Jas.* W. *Bk.*

V. *NYMPHÆACEÆ.*—The water lily tribe.

87. Nymphæa odorata. White pond lilly. June–Jul. *w.* Floating. ♃. pl. O. Mich. Slightly astringent.

88. Nuphar advena. Water lily. June. *y.* Floating or erect. ♃. pl. O. Properties similar to the last.

89. N. lutea, *Smith.* Yellow pond lily. Jul. *y.* Floating. ♃. Big Miami, O. Slightly astringent.

VI. *NELUMBONEÆ.*—The lotus tribe.

90. Nelumbium luteum. Water chinquapin. Chinquapin lily. Sacred bean. Jul. *w–y.* Generally floating. ♃. pl. O. Cin. Pickaway Plains. Mo. Ter. *Jas.*

VII. *HYDROPELTIDEÆ.*

91. Hydropeltis purpurea. Water target. Jul. *p.* Floating. ♃. pl. St. L. *Bk.* Demulcent, &c. Raf. Med. Fl. i.

92. Flœrkea uliginosa. False mermaid. Ap. *w.* ⊙. mh. Ky. *St.*

VIII. *PODOPHYLLEÆ.*—The may apple order.

93. Podophyllum peltatum. May apple. Mandrake. May. *w.* 1 f. ♃. ow. W. Cathartic. Big. and Bart. Bot. &c.
94. Jeffersonia diphylla. Twin leaf. Rheumatism root. M. *w.* 1 f. ♃. sw. W. Stimulant, diaphoretic, anti-rheumatic, antispasmodic. Chronic rheumatism especially. *Dr. I. G. Jones.*

IX. *CRUCIFERÆ.*—The cruciferous order.

It may be remarked that the Cruciferæ universally possess antiscorbutic and stimulant qualities, combined with a flavour more or less acrid.

95. Cheiranthes asper, *Nutt.* June. *y.* 12 to 18 i. ♂. Plains of the Missouri, near White river. *Nutt.*

96. Nasturtium officinale, *Brown.* Water cress. Jul. *w.* Stem decumbent. ♃. In water. W. *Bk.* A sallad.

97. N. palustre, *De Cand.* Marsh water cress. Jul. *y.* 18 i. ⊙. mh. W.

98. N. amphihium, *Brown.* Jul. *y.* 1 to 2 f. ♃. mh. O. W. Amphibious cress.

99. Barbarea vulgaris. Bitter winter cress. June. *y.* 12 to 18 i. ♃. Fields. O. Ky.

100 Arabis rhomboidea. Spring cress. May. *w.* 10 to 12 i. ♃. Woods. O. Ky.

101. A. hirsuta. Hairy tower mustard. June. *w.* 6 to 12 i. ♂. rs. bt. Upper Mississippi. *Houghton.* O.

102. A. lævigata. Smooth tower mustard. May. *w.* 1 to 2 f. rs. Wor. Cin. O.

103. A. canadensis. Sickle pod. June. *w.* 1 to 2 f. rs. W.

104. A. thaliana. Mouse ear cress. Common wall cress. Ap. *w.* 2 to 8 i. ⊙. Ky. *St.*

105. A. lyrata. Ap.–June. *w.* 8 to 12 i. ♂. Fields and hills. West. Va. *Paddock.* N. W. Ter. *Houghton.*

106. Cardamine virginica. American water cress. May–Jul. 6 to 18 i. ⊙. br. mh. O. Ky.

107. C. rotundifolia. Round leaved cress. Jul. *w.* 12 to 15 i. ♃. Wet grounds. Ky. *St.*

108. C. uniflora. Single flowered cress. Ky. *St.*

109. Dentaria diphylla. Tooth root. Trickle. Pepper root. May. *w.* 8 i. ♃. sw. Cin. O. Ky.

110. D. laciniata. Jagged leaved tooth root. May. pale *w.* 8 to 12 i. ♃. sw. W.

111. D. heterophylla. Dwarf tooth root. Jul. pale *p.* 2 to 4 i. ♃. Woods. West Penn. *Nutt.* West Va. *Paddock.*

112. Alyssum dentatum. May. *w.* 6 i. ♃. rs. Ky.

113. A. ludovicianum. Ap. *y.* ♃. Mo. Ter. *Nutt.*

114. Draba caroliniana. Ap. *w.* 2 to 4 i. ⊙. Side of mounds near St. L. *Bk.*

115. D. arabizans. June. *w.* ♂. rs. W. *Bk.*

116. Thalspi bursa-pastoris. Shepherd's purse. Ap.–Oct. *w.* 6 to 12 i. ⊙. cf. O. Ky.

117. T. arvense. Penny cress. June. *w.* 1 f. rb. Cin. O. Detroit. *Nutt.*

118. T. tuberosum. May. *r.* 4 to 5 i. West Penn. *Nutt.*

119. Cakile americana, *Nutt.* American sea rocket. Jul.–Oct. *p.* ⊙. Shores of the great lakes. *Nutt.*

120. Hesperis pinnatifida. Wild rocket. Jnne. *p.* 18 to 24 i. ♃. sw. W.

121. H. matronalis. Dame's violet. Garden rocket. ♃.—Shores of Lake Huron. *Dr. Todd.*

122. Sisymbrium officinale. Hedge mustard. June–Sept. *y.* 1 to 2 f. ⊙. rd. W.

123. S. canescens. Ap. *y.* 1 to 2 f. ⊙. Banks of the Miss. St. L. *Bk.*

124. Coronopus didyma. Swine's cress. Jul. 1 to 2 f. ⊙. Mo. Ter. *Nutt.*

125. Stanleya pinnatifida. May. *y*. 3 f. ♃. cr. Mo. Ter. *Nutt.* The most splendid of the cruciferæ.

126. Lepidium virginicum. Wild pepper grass. June. *w*. 12 to 18 i. ⚨. rd. cf. O. Ky.

127. Sinapis nigra. Black mustard. Common mustard. June. *y*. 2 to 4 f. ⊙. bt. Introduced. Seeds are stimulant, laxative, rubefacient, antiscorbutic.

128. Cochlearia armoracia. Horse radish. June. *w*. 2 f. ♃. Introduced. Stimulant, antiscorbutic.

X. *FUMARIACEÆ*,—The fumitory tribe.

129. Diclytra canadensis, *Goldie*. Turkey corn. Squirrel corn. May. Pale *r–w*. 8 i. ♃. sw. O. Flowers with an exquisite fragrance. Root two or three remote, yellow spheres, somewhat resembling kernels of Indian corn. Probably, secernant stimulant, diuretic, diaphoretic. Said by Dr. I. G. Jones to be a substitute for mercury in veneral complaints. Rad. Dic. can. ℥i. ter die, in inf. Externally also as a lotion. Applicable either to gon. or syph.

130. D. cucullaria, *De Cand.* Dutchman's breeches. April. 6 i. ♃. sw. W. Resembles Sp. 129 closely. Root a congeries of whitish, ovid tubers, immediately at the base of the stem,—very similar to the preceding in taste.

131. Corydalis aurea. Golden corydalis. M.–Ju. *y*. 8 to 12 i. ⊙. rs. bt. W.

132. C. fungosa. Climbing corydalis. Colic weed. June. *r–w*. ⚨. West Penn.

XI. *CAPPARIDEÆ*.—The caper tribe.

133. Polanisia graveolens, *Raf.* False mustard. Ju. *p–y*. 8 to 12 i. ♃. rb. O. Ky. Narcotic vermifuge.

134. Cleome serrulata. Aug. *p–w*. 3 to 4 f. ⊙. Mo. Ter. *Nutt.*

XII. *ANONACEÆ*.—The custard apple tribe.

135. Asimina triloba. Papaw. Custard apple. Ap. Dark *r*. 10 to 20 f. ♄. bt. W. Fruit large, rich, fragrant, and by some esteemed as a luxury.

XV. *MAGNOLIACEÆ*.—The magnolia tribe.

136. Magnolia glauca. Sweet bay. Beaver tree. Swamp laurel. M.–Ju. *w*. ♄. Louisville, *M'Murt*. Bark tonic. Flowers fragrant, stimulant, anti-rheumatic. Bart. i. 77. Big. ii. 67.

137. M. tripetala. Umbrella tree. June. *w*. 25 to 35 f. ♄. Mountain woods, &c. West Va. *Paddock*. Ky. *St.* Qualities like the last.

138. M. macrophylla. Giant-leaf magnolia. June. *w.* 30 to 35 f. ♄. Ky. *St.* Tenn. *Nutt.*
139. M. cordata. Heart leaf magnolia. May. *y.* 20 to 40 f. ♄. Ky. *St.*
140. M. acuminata. Cucumber tree. June. *b–y.* 70 f. ♄. Eastern part of Ohio. Bark tonic. The cones yield an anti-rheumatic, stimulating quality to alcohol.
141. M. auriculata. Ear-leaf magnolia. May. *y–w.* 30 to 40 f. ♄. Ky. *St.*
142. M. grandiflora. Big laurel. Magnolia. May. *w.* 60 to 80 f. ♄. Louisville, *M'Murt.*
143. Liriodendron tulipifera. Tulip tree. White wood. Tulip bearing poplar. July. *y–g.* 60 to 90 f. ♄. W. Tonic, antiseptic. Bart. Med. Bot. I. 91.

XXI. *LAURINEÆ.*—The cinnamon tribe.

144. Laurus benzoin. Benzoin. Spice wood. Fever bush. Ap. *y.* 6 to 10 f. ♄. Woods. W. Aromatic, stimulant, tonic. Bart. ii. 91.
145. Laurus sassafras. Sassafrass. April. *y.* 5 to 50 f. ♄. Woods. W. Stimulant, demulcent, diaphoretic.

XXII. *BERBERIDEÆ.*—The barberry tribe.

146. Berberis vulgaris. Barberry. April. *y.* 4 to 6 f. ♄. rs. Tenn. *Nutt.* Acid, astringent.
147. Leontice thalictroides, *Linn.* Blue cohosh. Poppoose root. Ap. *g–y.* 1 f. ♃. sw. O. Ky. Bitter, diuretic, preparatory parturient. The root.

XXIII. *MENISPERMEÆ.*—The cocculus tribe.

148. Menispermum canadense. Moonseed. Yellowparilla. July. *g–y.* Vine. ♄. bt. O. Ky. Tonic, diuretic, alterative. The root.

149. M. similacinum. July. *y.* Vine. ♄. Ky. *St.*

150. M. lyoni. July. ♃. Ky and Tenn. *Lyon.*

XXIV. *MALVACEÆ.*—The mallow tribe.

Generally possess mucilaginous, demulcent properties.

151. Sida abutilon. Indian mallows. Jul. *y.* 4 f. ⊙. rd. W.

152. S. dioica. Sept. *w.* 4 to 7 f. ♃. md. Miami country, O.

153. S. spinosa. July. *y.* 1 to 2 f. ⊙. rd. W.

154. S. alcæoides, Jul. ♃. Stony fields of Ky. and Tenn. *Pursh.*

155. Nuttallia digitata. May. *r.* 3 to 4 f. ♃. Pawnee villages.

156. Hibiscus militaris. Martial hibiscus. Aug. *w–y.* 4 to 6 f. ♃. Wet woods. O. Ky.

157. H. moscheutus. Aug. *w–p.* 4 to 6 f. Swamps. Ky. *St.*
158. H. pulustris. Marsh hibiscus. Aug. *p.* 3 to 4 f. ♃. mh. Louisville. *M'Murt.* It is probable that Dr. M'Murtrie mistook one of the preceding for this. See his picture of Louisville. Appendix.
159. H. grandiflorus. Large flowering hibiscus. Aug. *r.* 5 to 7 f. ♃. Arkan. *Jas.*
160. Malva rotundifolia. Low mallows. June–Oct. Whitish pink. Prostrate. ♃. rd. W. Naturalized. Medicinal.

XXIX. *TILIACEÆ.*—The linden tribe.

161. Tilia glabra. Bass-wood. Linn. Linden. June. *w.* 60 f. ♄. Woods. Emollient.
162. T. pubescens. Crop-ear bass-wood. June. *w.* 60 f. ♄. Riv. banks. W.
163. T. hyterophylla. Various leaved bass-wood. June. ♄. Banks of the Ohio and Mississippi. *Pursh.*

XXXVI. *HYPERICINEÆ.*—The tutsan tribe.

164. Hypericum pyrimidatum. Giant hypericum. Jul. *y.* 3 to 6 f. ♃. Wor. O.

165. H. prolificum. June–Aug. *y.* 1 to 3 f. ♄. dp. O. Ky.

166. H. kalmianum. Kalm's shrubby hypericum. Jul. *y.* 3 to 5 f. ♄. Lake Huron. *Eat.*

167. H. galioides. Jul. *y.* 2 f. ♄. Darby Plains, O.

168. H. perforatum. St. John's wort. Jul. *y.* 1 f. ♃. Fields. W. Introduced? Healing, pectoral.

169. H. corymbosum. Level-top hypericum. Jul. *y.* 18 to 24 i. ♃. W.

170. H. parviflorum. Low centaury. Small flowered hypericum. Jul. *y.* 6 to 10 i. ♃. wp. O. Ky.

171. H. densiflorum. Many flowered hypericum. Jul. *y.* 2 f. ♄. Darby Plains, O.

172. H. virginicum. Virginian hypericum. Aug.–Sept. *y.* 2 f. ♃. Wet woods. Wor. O.

173. H. canadense. Canadian hypericum. June–Aug. *y.* 6 to 12 i. ⊙. Detroit. *Eat.*

174. H. rosmarinifolium. Rosemary-like hypericum. Jul. *y.* 2 to 4 f. ♄. Low grounds. St. L. *Bk.*

175. H. frondosum. Jul. *y.* ♄. rs. Ky. and Tenn. *Pursh.*

176. H. dolabriforme. Jul. *y.* ♃. Dry hills. Ky. *Michx.*

177. H. triplinerve. Aug. *y.* ♃. Banks of the Ohio. *Michx.*

178. H. sphærocarpum. *y.* ♃. *Michx.*

179. H. procumbens. *y.* Dry hills. Ky. *Michx.*

180. Ascyrum crux-andreæ. St. Peter's wort. Jul. *y.* 1 f. ♃. Sandy fields, Ky. *St.*

181. A. stans, *Michx,* Jul.–Aug. *y.* 1 to 2 f. ♃. Louisville. *M'Murt.*

182. Sarotha gentianoides. Pine weed. Nit weed. Orange grass. June. *y–p.* 4 to 8 i. ⊙, Ky. *St.*

XXXVIII. *SAXIFRAGEÆ.*—The saxifrage order.

All plants of this order are more or less astringent.

183. Saxifraga pennsylvanica. Large saxifrage. Water saxifrage. May–June. *y–g.* 2 to 5 f. ♃. md. mh. Wor. O.

184. Saxifraga virginiensis. Early saxifrage. Ap.–May. *w.* 4 to 12 i. ♃. rs. W.

185. Heuchera americana. Alum root. June. *r.* 2 to 3 f. ♃. rs. W. Powerfully astringent. Bart. Med. Bot. ii. 159.

186. H. pubescens. May–June. *r–y.* 2 f. ♃. Ky. *St.*

187. H. cauluscens. Jnne. *w.* ♃. Ky.

188. H. acerifolia. ♃. Ky.

189. Chrysosplenium oppositifolium. Golden saxifrage. Water carpet. Ap. *g.* Creeping. ♃. Springs, &c. Near Chillicothe, O.

190. Mitella diphylla. Currant leaf. False sanicle. May. *w.* 8 to 10 i. ♃. rs. W.

191. M. cordifolia. June. 6 to 8i. ♃. Detroit. *Eat.*

192. Tiarella cordifolia. Mitre wort. May. *w.* 8 to 10 i. ♃. sw. rs. Ky. *St.*

193. Parnassia americana. Flowering plantain. Parnassus grass. Sept. *w.* 12 to 13 i. ♃. wp. O. Mich.

194. Itea virginica. June. *w.* 4 to 8 f. ♄. Louisville. *M'Murt.*

XLII. *HAMAMELIDEÆ.*—The witch-hazel tribe.

195. Hamamelis virginica. Witch-hazel. Nov. *y.* 6 to 12 f. ♄. Woods. O. Ky. Astringent, sedative.

XLIII. *PHILADELPHIÆ.*—The mock orange tribe.

196. Philadelphus hirsutus. Hairy syringa. *w.* ♄. Tenn. *Nutt.*

XLV. *GROSSULACEÆ.*—The currant tribe.

197. Ribes hirtellum. Wild gooseberry. May. *g.* 2 to 3 f. ♄. rs. Wor. O.

198. R. oxycanthoides. Smooth gooseberry. May. *y.* 3 f. ♄. rs. Louisville. *M'Murt.*

199. R, cynosbati. Prickly gooseberry. Ap.–June. *w–g.* ♄. Ky. *St.*

200. R. floridum. Wild black currant. Ap.–May. *y.* 3 to 4 f. ♄. Hedges. Covington, Ky.

201. R. albinervum. Ap.–May. *g–y.* ♄. Sources of the St. Croix river. *Houghton.*

### XLVI. *CACTEÆ.*—The Indian fig tribe.

202. Cactus opuntio. Prickly pear. June. *y.* ♄. Sandy banks, Ky. Ill.

203. C. ferox. July. *y–r.* ♃. Arid plains. Mo. Ter. *Nutt.*

204. C. viviparus. July. *r.* ♃. Mo. Ter. *Nutt.*

205. C. mamillaris. Mo. Ter. *Nutt.*

### XLVII. *ONAGRARIÆ.*—The evening primrose order.

206. Epilobium spicatum. Willow herb. July. *p.* 3 to 5 f. ♃. New meadows. W. *Bk.* Root emollient, slightly astringent, anti-dysenteric.

207. E. coloratum. July. *p.* 3 to 4 f. ♃. wp. md. W. *Bk.* Ky. *St.*

208. E. palustre. Marsh willow herb. Aug.–Sept. Pale *r.* 2 to 3 f. ♃. wp. br. O.

209. E. lineare. July. *w–r. w.* 1 to 2 f. wp. O. Two miles south of Columbus, eight miles east of Dayton.

210. Gaura biennis. Virginian loosestrife. July–Aug. *r.* 2 to 3 f. ♂. dp. O. Ky. *St.* Near St. L. *Bk.*

211. G. mollis. July–Aug. *r.* 3 f. dp. O?

212. Œnothera biennis. Evening primrose. Scabish. Tree primrose. June–Sept. *y.* 3 to 5 f. ♂. Fields, &c. W.

213. Œ. muricata. July–Aug. *y.* 1 to 2 f. ♂. Fields, O. Ky.

214. Œ. grandiflora. Large flowering evening primrose. July–Aug. *y.* 2 to 3 f. ♂. Fields. Ky. *St.* Figured in Bart. Fl. N. A. i. 20.

215. Œ. tetragona. ♃. Ky? *St.*

216. Œ. sinuata. June. *y.* 1 to 6 i. ♃. Mound near St. L. *Bk.*

217. Œ. albicaulis. White-stem evening primrose. July. *w.* 3 f. ♃. Plt. Mo. Ter. *Nutt.*

218. Œ. pinnatifida. June. *w.* 6 to 24 i. ♂. White river, M. Ter. *Nutt.*

219. Œ. cæspitosa. Jul. *w–r.* ♃. White riv.

220. Œ. serrulata. June. *y.* Plt. Mo. Ter. *Nutt.*

221. Œ. macrocarpa. Aug. *y.* ♂. Mo. near St. L. *Nutt.* Near Lead mines. *Bk.*

222. Œ. viscosa. Louisville. *M'Murt.*

223. Œ. fruticosa. Sundrops. Shrubby œnothera. Jul. *y.* 12 to 18 i. ♃. ♄? dp. Near Columbus, O.

224. Jussieua leptocarpa. *y.* ⊙. Mo. Ter. *Nutt.*

225. J. subacaulis. June. *y.* ♃. Mo. Ter. *Lewis.*

226. Ludwigia alternifolia. Seed box. Jul. *y.* 1 to 3 f. ♃. mh. O. Ky.

227. L. decurrens. Aug. *y.* 2 f. ☉. Louisville. *M'Murt.*
228. Isnardia palustris. Water purslane. June. *g.* Creeping. ♃. Mud, &c. O. Ky.

XLVIII. *HALORAGEÆ.*—The hippuris tribe.

229. Hippuris vulgaris. Marestail. Aug. *y–g.* 12 to 18 i. ♃. pl. Ky. *St.*

230. Callitriche verna. Water chickweed. May–Aug. *w.* Floating. 2 to 3 feet long. Shallow streams. Ky. *St.*

231. Myriophyllum verticillatum. Water milfoil. Jul. 18 i? ♃. pl. Plt. *Jas.* Cin. O.

XLIX. *CIRCÆACEÆ.*—Order of the enchanter's night shade.

232. Circæa lutetiana. Enchanter's night shade. Jul. *w.* 1 to 2 f. ♃. sw. W.

233. C. alpina. Jul. *r–w.* 4 to 8 i. ♃. sw. Wor. O.

LI. *LOASEÆ.*

234. Bartonia ornata. *Nutt.* Barton's flower. *y–w.* 2 to 4 f. ♃ ? sr. Mo. Ter. *Nutt.* Figured: Bart. Fl. N. A. iii. 29.

235. B. nuda. *y–w.* ♃. Mo. Ter, near the Great Bend of the Missouri. *Nutt.*

236. Mentzelia aurea. *y.* 12 i. Near the Lead Mines of St. Louis. Plt. riv. *Nutt.*

LII. *SALICARIÆ.*—The grass poley tribe.

237. Cuphea viscosissima. Wax bush. Sept. *p.* 12 to 18 i. ☉. W. Figured. Bart. Fl. N. A. i. 62.
238. Lythrum hyssopifolium. Dwarf grass poley. Aug. *p.* 12 to 24 i. ♃. wp. W.
239. L. alatum. Jul. *p.* 2 to 3 f. ♃. mh. St. L. *Bk.*
240. L. virgatum. Jul. *p.* ♃. Louisville. *M'Murt.*
241. Decodon verticillatum, *Elliott.* Grass poley. Swamp willow herb. Aug. *p.* 2 to 4 f. ♃. mh. or wet places. Ohio near Dayton. Louisville. *M'Murt.* Ky. *St.*
242. Ammannia humilis. Aug. *b.* 4 to 8 i. ☉. Damp ground. Ky. *St.*

LIV. *MELASTOMACEÆ.*—The meadow beauty order.

243. Rhexia virginica. Meadow beauty. Deer grass. Jul. *p.* 1 to 2 f. ♃. Ky. *St.* Mo. *Bk.* Figured. Bart. Fl. N. A. i. 12.

244. Rhexia mariana. Jul. *w—r.* 1 to 2 f. ♃. Ky. *St.* Fig. Bart. Fl. N. A. i. 96.

LIX. *ELÆAGNEÆ.*—The oleaster tribe.

245. Elæagnus argentea. The silver oleaster. Ju. ♄. 8 to 12 f. Mo. Ter. *Nutt.*

246. Shepherdia canadensis, *Nutt.* Sea buck thorn. Jul. 6 to 8 f. ♄. Mich. *Eat.*

247. S. argentea. Rabbit berry. 12 to 18 f. ♄. Plt. *Jas.*

LXII. *ARISTOLOCHIÆ.*—The birthwort tribe.

248. Aristolochia sipho. Birthwort. Dutchman's pipe. June. Flowers brown. Vine 30 to 40 f. ♄. rs. Pittsburgh. Louisville, *M'Murtrie.*
249. A. serpentaria. Virginia snakeroot. June. Brownish *p.* 8 to 12 i. ♃. sw. O. Ky. Stimulant, tonic, diaphoretic, diuretic. Big. Med. Bot. iii. 82. Bart. Med. Bot. ii. 41.
250. Asarum canadense. Wild ginger. Coltsfoot snakeroot. Ap. *p.* 3 to 4 i. ♃. ow. sw. W. Aromatic stimulant, tonic, diaphoretic. Big. Med. Bot. i. 179. Bart. Med. Bot. ii. 85.

LXIV. *SANTALACEÆ.*—The sanders-wood tribe.

251. Nyssa multiflora. Sour gum. Black gum. Ju. *g.* 30 to 50 f. ♄. Woods. W.
252. N. biflora, *Walt.* Tupelo tree. Swamp horn bean. Ju. 30 to 50 f. ♄. Ky. *St.*
253. N. tomentosa. May. ♄. Ky. *St.*
254. Thesium umbellatum. Bastard toad-flax. Jul.–Aug. *w.* 8 to 12 i. ♃. rs. W.

LXV. *THYMELEÆ.*—The mezereum tribe.

255. Dirca palustris. Moose wood. Leather wood. Ap. *y.* 2 to 4 f. ♄. Woods. O. Stimulant, acrid, vesicatory.

LXXII. *SANGUISORBEÆ.*—The burnet tribe.

256. Sanguisorba canadensis. Burnet saxifrage. Aug.–Oct. *w.* 2 to 4 f. ♃. wp. Dayton, Columbus, O. Slightly astringent and tonic.

LXXIII. *ROSACEÆ.*—The rose tribe.

§ 1. Potentilleæ.—*Strawberry like.*

257. Potentilla canadensis. Cinquefoil. Five-finger. Ap.–

May. *y.* Trailing. ♃. ow. O. Ky. Astringent, &c. One of Thompson's medicinal plants.

258. P. simplex. Erect cinquefoil. May–Aug. 8 to 12 i. ♃. ow. Wor. O. St. L. *Bk.*

259. P. norwegica. Norway cinquefoil. June–Aug. *y.* 8 to 18 i. ⊙. O.

260. P. hirsuta. Hairy cinquefoil. Jul. *y.* ⊙. Wor. O.

261. P. fruticosa. Shrubby cinquefoil. June–Sept. *y.* 2 f. ♄. wp. Mich. O.

262. P. pennsylvanica. June. *y.* 1 to 2 f. ♃. Detroit. *Eat.*

263. P. argentea. Silver five-finger. Jul. *w–y.* 4 to 10 i. ♃. Prairies near St. L. *Bk.*

264. P. supina. June–Aug. *y.* ⊙. Creeping on the ground. Mich. *Eat.* W. *Bk.*

265. Comarum palustre. Marsh five-finger. Jul. *p.* 12 to 18 i. ♃. mh. bogs. Louisville, *M'Murtrie.* An active astringent. *Eat.*

266. Fragaria virginiana. Wild strawberry. May. *w.* ♃. O. Ky.

267. F. canadensis. Mountain strawberry. Ap.–May. *w.* ♃. Mich. *Eat.* Ky. *St.*

268. Dalibarda repens. Spice root. False violet. June. *w.* Creeping. ♃. Wor. O.

269. D. fragaroides. Dry strawberry. May. *y.* 3 to 4 i. ♃. Woods. Wor. Cin. O.

270. Agrimonia eupatoria. Agrimony. Jul. *y.* 2 f. ♃. wp. ow, if damp. O. Ky. Astringent, corroborant, deobstruent, Hoop. Med. Dic. Raf. Med. Fl., &c.

271. A. parviflora. Small flowered agrimony. Aug. *y.* 1 to 2 f. ♃. ow, if dry. O. Ky. Properties like the last.

272. A. suaveolens. Jul. *y.* 5 f. ♃. Borders of marshes. St. L. *Bk.*

273. Geum strictum. Upright avens. Yellow avens. Aug. *y.* 2 f. ♃. West Va. *Paddock.*

274. G. virginianum. Avens. Virginian avens. June–Jul. *w.* 2 f. ♃. sw. ow. W. Medicinal. Astringent.

275. G. album. White avens. June–Jul. *w.* 2 f. ♃. Prairies 12 miles E. Wor. O.

276. G. ciliatum. Jul. *y—p.* 12 to 18 i. md. O.

277. G. rivale. Purple avens. Water avens. Evinroot, in New York. May–June. *p.* 18 i. Said to grow in the northern part of Ohio. Astringent, tonic. In New York the root sometimes substitutes coffee.

278. Rubus strigosus. Red raspberry. May. *w.* 3 to 5 f. ♄. O. Mildly astringent. The leaves are used.

279. R. occidentalis. Black raspberry. Wild raspberry.

Monthly raspberry. May–Jul. May–Oct. *w.* 2 to 5 f. ♄. ow. W.

280. R. villosus. Blackberry. June. *w.* 4 to 6 f. ♄. ow. &c. W. Astringent. See Big. Med. Bot.

281. R. trivialis. Low blackberry. Dewberry. May–June. *w.* Mostly trailing. ♄. ow. &c. O. Ky. Properties similar to the last.

282. R. idæus. Raspberry. June. *w.* 3 to 5 f. ♄. Fruit red. ow. Ky. St. Mo. Ter. *Jas.* Britain. *Loudon.*

283. R. parviflorus. Small flowering bramble. *w.* ♄. Mich. *Eat.*

284. R. odoratus. Flowering raspberry. Wild mulberry. Ju. *p.* 3 to 4 f. ♄. rs. O. Ky.

285. Sibbaldia procumbens. Ap. *y.* Procumbent. ♃. Mo. Ter. *Nutt.*

§ 2. Roseæ.—*Rose like.*

286. Rosa rubifolia. Climbing rose. Ohio multiflora. Jul. *r,* changing to *w.* The rose referred to is very common on the borders of the Ohio prairies. It sometimes appears as a shrub 5 or 6 feet high; more frequently it climbs as a woody vine to the height of 15 or 20 feet. Its magnificent appearance when in full bloom is almost unequalled. O. Ky. Lake shores.

287. R. blanda. Jul. *r.* 5 to 8 f. ♄. Mar. O.

288. R. rubiginosa. Sweet-briar. Eglantine. July. Pale *r.* 3 to 10 f. ♄. Hedges, &c. Ky. O.

289. R. parviflora. Small-flowered rose. Ju.–July. *r.* 3 f. ♄. Woods. W.

290. R. carolina. June–Jul. *r.* 3 to 8 f. ♄. Swamps. Mo. *Beck.*

291. R. lucida. Jul. *r.* 3 to 4 f. ♄. Louisville, *M'Murt.*

§ 3. Spiræaceæ.—*Meadow-sweet like.*

292. Spiræa salicifolia. Willow-leaved spiræa. Jul. *w.* 2 to 4 f. ♄. md. wp. W.

293. S. tomentosa. Hard-hack. Jul.–Aug. *p.* 2 to 3 f. ♄. Ky. *St.*

294. S. opulifolia. Nine-bark. June–Jul. *w.* 3 to 6 f. ♄. sr. Wor. O. Ky.

295. S. betulifolia. Birch-leaved spiræa. June. *r.* 1 f. ♄. Ky. *St.*

296. S. aruncus. Goats-beard. Steeple-weed. June. *w.* 2 to 6 f. ♃. Mar. O. Ky.

297. S. lobata. Meadow-sweet. Pride of the prairie. June. *r.* 2 to 4 f. ♃. wp. O.

298. Gillenia trifoliata. Indian physic. Gillenia. June. *w.* 2 to 3 f. ♃. sw. Morgantown, Va. *Paddock.* Emetic, emeto-cathartic, diaphoretic. Big. Med. Bot. iii. 10. Bart. Med. Bot. i. 65.
299. G. stipulacea. Western gillenia. American ipecac. Ju.–Jul. *w.* 2 to 4 f. ♃. Woods. W. Properties similar to the last. Fig. Bart. Med. Bot. i. 71.

LXXIV. *POMACEÆ.*—The apple tribe.

300. Aronia arbutifolia. Red choak-berry. May–June. *w–r.* 2 to 4 f. ♄. Louisville, *M'Murt.*

301. A. botryapium. Shad bush. June berry. Service berry. Ap. *w.* 8 to 30 f. ♄. O. Ky.

302. A. melanocarpa. Black choak-berry. May–June. *w.* 4 to 6 f. ♄. Bogs, &c. Ky. *St.*

303. A. sanguinea. Bloody choak-berry. May. *w.* 4 to 6 f. ♄. W. *Pursh.*

304. A. alnifolia. Alder-leaved service berry. May. 4 to 5 f. ♄. Mo. Ter. *Nutt.*

305. Crategus coccinea. Thorn bush. Red haw. May. *w.* 5 to 15 f. ♄. Woods. O. Ky.

306. C. crus-galli. May. *w.* 5 to 20 f. ♄. W.

307. C. populifolia. Ap. *w.* ♄. Ky. *St.*

308. C. pyrifolia. Pear-leaf thorn. June. *w.* ♄. Michigan, *Beck.* O.

309. C. punctata. Thorn tree. May. *w.* ♄. O.

310. C. glandulosa. May. *w.* ♄. O.

311. C. spathulata. Ap. *w.* 10 to 12 f. ♄. O.

312. C. apiifolia. Ap. *w.* 4 to 12 f. ♄. O.

313. Sorbus americana. Mountain ash. May. *w.* 10 to 20 f. ♄. Near Lake Huron, *Houston.*

314. Pyrus coronaria. Crab apple. May. Pale *r.* 15 to 20 f. ♄. W.

LXXV. *AMYGDALEÆ.*—The almond tribe.

315. Prunus virginiana. Black cherry. Wild cherry. May. *w.* 30 to 60 f. ♄. Woods. W. Tonic, anodyne. Big. Med. Bot.
316. P. pennsylvanica. Red cherry. Wild red cherry. Ap.–May. *w.* 8 to 20 f. ♄. Ky. *St.*
317. P. serotina, *De Cand.* Choke cherry tree. June. *w.* 20 to 60 f. ♄. Ky. *St.*
318. P. depressa. Sand cherry. May. *w.* 2 to 6 f. ♄. River banks, Ky. *St.* Fruit black, agreeable.
319. P. americana. Meadow plum. Wild red plum. Yellow plum, *Beck.* May. *x.* 10 to 12 f. ♄. md. Prairies, &c. O.

320. P. chicasa. Summer plum. May. *w.* 10 to 15 f. ♄. Ky. *St.* Ark. *Jas.*

LXXVII. *LEGUMINOSÆ.*—The pea tribe.

321. Baptisia alba. Prairie indigo. Jul. *w.* 2 to 4 f. ♃. dp. Central Ohio. Medicinally a substitute for B. tinctoria. *Dr. Jones.*
322. B. cœrulea. Blue flowered indigo. Spiked indigo weed. Jul. *b.* 2 to 3 f. rb. ♃. Ky. Cin. O.
323. B. tinctoria. Wild indigo. Jul.–Aug. *y.* 2 to 3 f. ♃. Ky. *St.* Astringent, antiseptic, cathartic, emetic, stimulant. Beach. Mat. Med. Raf. Med. Fl.
324. B. bracteata. Ap. *w.* 2 f. ♃. St. L. *Nutt. Jas.*
325. Sophora sericea. *w.* ♃. 1 f. Plt. *Jas.* Mo. Ter. *Nutt.*
326. Medicago lupulina. Hop medick. None-such. Ju.–Aug. *y.* Procumbent. ⊙. Throughout the U. S. *Bk.* Introduced.
327. Psoralea eglandulosa. June. *p.* 12 to 18 i. ♃. O. Ky. Tenn. *Nutt.*
328. P. cuspidata. ♃. Mo. Ter. *Nutt.*
329. P. esculenta. Bread root. June. *b.* 12 to18 i. ♃. Various part of Mo. Ter. Lead Mines near St. Louis, *Nutt.* The root is an important article of diet to the Western Indians.
330. P. incana. Jul. *b.* 12 i. ♃. Open plains, Mo. Ter. *Nutt.*
331. P. lanceolata. Aug. *w–b.* 1 f. ♃. Plt. *Nutt.*
332. P. onobrychis. 3 to 5 f. Near St. L. *Nutt.*
333. P. canescens. June. *y.* 2 to 3 f. ♃. St. L. *Jas.*
334. P. jamesii. 4 i. St. L. *Jas.*
335. Trifolium repens. White clover. May–Oct. *w.* 6 to 12 inches long, creeping. ♃. Valuable in pastures.
336. T. procumbens. Yellow clover. June. *y.* Procumbent, 3 to 6 inches long. Dry fields, Ky. *St.* Mar. O.
337. T. stoloniferum. Running buffalo clover. Ju. *w.* Creeping, 4 to 8 inches long. ♃. Fields, ow., &c. O. Ky.
338. T. reflexum. Buffalo clover. June. *r.* Procumbent, 12 to 18 i. Dry hills, Ky. *St.* Wor. O. Great Lakes.
339. T. pratense. Red clover. May. *r.* 2 to 3 f. ♃. md. Introduced. W. A valuable artificial grass.
340. Lespedeza capitata. July–Aug. *p.* 2 to 3 f. ♃. dp. ow. O. Ky.
341. L. violacea. Jul. *p.* 1 to 2 f. ♃. dp. Ky. Mar. O.
342. L. procumbens. Aug.–Sept. *p–y.* Procumbent, 2 to 4 f. ♃. Sandy woods, Ky. *St.*

343. L. prostrata. Aug. *p.* ♃. Sandy soils, Ky. *St.*

344. L. augustifolia. Sept. *w–p.* 3 to 4 f. ♃. Sandy soils, ow. dp. Col. Mar. O.

345. L. polystachia. Aug.–Sept. *r–w.* 2 to 4 f. ♃. dp. ow. O.

346. L. divergens. Jul. *p.* 1 to 2 f. ♃. dp. Darby Plains, Cin. O.

347. L. reticulata, *Bk.* Bush clover. Aug. *p.* 2 f. ♃. Dry woods, Ill. *Bk.*

348. Hedysarum canadense. Canadian trefoil. Jul. *p.* 3 to 4 f. ♃. dp. ow. W.

349. H. lævigatum. Smooth trefoil. Aug. *p.* 3 to 4 f. ♃. dp. Woods. O.

350. H. rotundifolium. Round leaved trefoil. Aug. *p.* Prostrate, 2 to 3 f. long. ♃. rs. O. Ky.

351. H. paniculatum. Aug. *p.* 3 f. ♃. ow. O.

352. H. rigidum. Rigid trefoil. Aug. *p.* 3 f. ♃. Mar. O.

353. H. acuminatum. Broad-leaf trefoil. Jul.–Aug. *p.* 1 to 2 f. ♃. sw. Ky. O.

354. H. nudiflorum. Naked-flowered trefoil. Aug. *p.* 1 to 2 f. ⊙. sw. Ky. O.

355. H. viridiflorum, *Bk.* Green-flowered trefoil. Jul. *g–p.* 3 to 4 f. ♃. sw. Ky. *St.*

356. H. aikinianum, *Bk.* Aikin's trefoil. Jul.–Aug. *r–p.* 3 f. ♃. ow. O. Ky.

357. H. glutinosum. Sticky trefoil. Jul. *p.* ♃. Ky. *St.*

358. H. pauciflorum. Few-flowered trefoil. *w.* ♃. Decumbent. W.

359. H. boreale, *Nutt.* Alpine trefoil. June–Jul. *p.* ♃. Mountains, &c. Mo. Ter. *Nutt.*

360. H. glabellum. Jul.–Sept. *p.* 1 to 2 f. ♃. Damp woods, one mile E. from Monroe, O. Detroit, *Eat.* Perhaps the most elegant plant of the genus.

361. H. obtusum. Blunt-leaf trefoil. Aug. *p–g.* 1 to 2 f. ♃. ow. dp. Mi. O.

362. H. strictum. Branchless trefoil. Jul. *p.* 2 to 4 f. ♃. dp. Middletown, O.

363. H. marilandicum. July–Aug. *p.* ⊙. Dry fields and woods. Louisville, *M'Murt.*

364. Galega virginiana. Goat's rue. Jul. *r. y. w.* 1 f. ♃. Barrens. Throughout U. S. *Bk.*

365. G. hispidula. May. *r.* 2 f. ♃. Ky? *St.*

366. Astragalus canadensis. Milk vetch. June. *y.* 2 f. ♃. Banks of streams. W.

367. A. missouriensis, *Nutt.* May. *p.* 6 i. Hills. Mo. Ter. *Nutt.*

368. A. galegoides. *w.* 2 f. Mo. Ter. *Nutt.* Near Zanesville, O?

369. A. hypoglottis. May. Mo. Ter. *Nutt.*
370. A. laxmanni. June. *b.* 1 f. ♃. Hills, Mo. Ter. *Nutt.*
371. A. carnosus. June. *b–p.* 2 f. Plains. Mo. Ter. *Nutt.*
372. A. gracilis. May. *p.* 2 f. ♃. Plains, Mo. Ter. *Nutt.*
373. Stylosanthes elatior. Pencil flower. Aug. *y.* 9 to 10 i. ♃. Louisville, *M'Murt.*
374. Petalostemon violaceum. Aug. *r–p.* ♃. Ky. *St.* Mo. Ter. *Nutt.*
375. P. candidum. Jul. *w.* ♃. Tenn. Ill. Mo. *Pursh.* Ky. *St.*
376. P. macrostachyum. *w.* 2 f. ♃. Plt. *Jas.*
377. Dalea aurea. Jul. *y.* 2 f. ♃. Gravelly hills, Mo. Ter. *Nutt.*
378. D. laxiflora. Jul. *w.* 6 f. ♃. Prairie du Chien, *Nutt.*
379. D. formosa. *p.* ♄. Plt. *Jas.*
380. D. alopecurioides. Jul. *b.* 1 f. ☉. Mississippi River, *Pursh.*
381. Phaca villosa. Jul. *y.* ♃. Hills, Mo. river, *Nutt.*
382. P. triphylla. May. ♃. Mo. Ter. *Nutt.*
383. Orobus dispar. Bitter vetch. June. *w–y.* ♃. Plt. *Jas.*
384. O. longifolius, *Nutt.* May. *r.* ♃. Sandy hills, Mo. Riv. Plt. *Nutt. Jas.*
385. Oxytropis lambertii. June. *p.* ♃. Plt. *Nutt. Jas.*
386. Thermia rhombifolia. March? *y.* 8 to 12 i. ♃. Plt. *Nutt. Jas.*
387. Glycyrrhiza lepidota. American liquorice. Jul. *w.* 3 to 5 f. ♃. bt. St. L. *Bradbury.* Mo. Ter. *Nutt. Jas.* Expectorant, demulcent. The root probably a substitute for the officinal liquorice. Disp. Wood and Bache. 322.
388. Trigonella sericea. American fenugreek. Jul. *w–r.* 1 f. ☉. bt. Plt. *Nutt.* Medicinal? Duncan's Disp. 319.
389. Amorpha fruticosa. Jul. 6 f. ♄. Ky. *St.* Plt. *Jas.*
390. A. nana. May. *p–b.* 9 i. ♄. Grassy, woodless hills. Plt. *Nutt.*
391. A. canescens. Lead plant. Mineral plant. July? *b.* 18 i. ♄. St. L. *Nutt.* Plt. *Jas.* This plant is said to grow only in the immediate neighborhood of lead mines.
392. Virgilia lutea. Virgil's tree. June. *y.* Tall tree. *St.* Ky. *St.* Tenn. *Nutt.* The bark affords a yellow dye. *Eat.*
393. Robinia pseudacacia. Locust tree. Black locust. False acacia. May. *w.* 30 to 50 f. ♄. bt. Woods. W.
394. Clitoria mariana, *Lin.* July–Aug. *b.* Climbing. ♃. bt. &c. O?
395. Clitoria virginiana. Aug. *p.* Climbing. ♃. Hedges.

Ky. *St.* Flowers larger than those of any other North American plant of this order.

396. Amphicarpa monoica. Jul. *b. w.* Twining. 3 to 4 feet long. ⊙. ow. O. Ky.
397. A. comasa. Jul. *p.* Prostrate and climbing. 3 to 5 f. ♃. Prairie near Middletown, O.
398. A. sarmentosa. Aug. *w.* Prostrate. ow? O?
399. Apios tuberosa. Ground-nut. Jul. *p.* Twining, 2 to 6 f. ♃. bt. ow. &c. O. Ky. Root tuberous and nutritious.
400. Phaseolus perennis. Wild bean. Jul. *p.* Twining. ♃. ow. W. *Bk.* Ky. *St.*
401. Strophostyles helvolus. July–Aug. *p.* Climbing or prostrate. ♃. Sandy fields. W. *Bk.*
402. S. diversifolius. Aug. *p.* Prostrate, 3 to 4 f. ⊙. Prairie near Hamilton, O. Woods. *Bk.*
403. Wistaria frutescens. Wistar's vine. *b–p.* Climbing. ♄. Ill. *Eat.*
404. Lupinus perennis. Wild lupine. May. *b.* 1 f. ♃. W. *Bk.* Louisville, *M'Murt.*
405. L. pusillus. Stinted lupine. June. *b.* 4 to 6 i. ⊙. or ♂. White River, Mo. Ter. *Nutt.*
406. Lathyrus decaphyllus. *p.* ♃. Plt. *Jas.* Grassy plains, Mo. Ter. *Nutt.*
407. L. venosus. Vein-leaved vetchling. July–Aug. *p.* ♃. wp. With grasses, &c. Mich. *Eat.* O.
408. Vicia americana. American vetch. May–June. *p.* Seldom erect. ♃. rb. sw. &c. O. Mo. Ter. W.
409. V. cracca. Tufted vetch. Aug. *p–w.* ♃. md. Ky. *St.*
410. V. caroliniana. Carolinian vetch. May–June. *w.* Climbing. ♃. Mountains, &c. Mich. *Eat.*
411. V. sativa. Tare. Common vetch. June. *b.* 1 to 2 f. ⊙. cf. Intr. W?
412. Pisum maratimum, *Lin.* Beach pea. May–July. *b–p.* ♃. Shores of the Great Lakes, *Nutt.*
413. Darlingtonia brachyloba. Darlington's plant. ♃. Ky. &c. *Eat.* Prairies, Ill. Ky. *Pursh.*
414. D. glandulosa. ♃. Tenn. *Eat.* Banks of Tenn. and Miss. *Pursh.*
415. Schrankia uncinata. Sensitive briar. Jul. *p.* 2 to 3 f. ♃. Plt. *Jas.* Near Potosi, Mo. *Bk.*
416. Cassia marilandica. American senna. Wild senna. July–Aug. *y.* 3 to 4 f. ♃. bt. W. Purgative. Big. Med. Bot. i. 166.
417. C. chamæcrista. Prairie senna. Partridge pea. July–Aug. *y.* 1 to 2 f. ⊙. dp. Sandy places. O. Ky.

Abundant on Darby Plains, three miles S. from Milford. The leaves of this plant are probably superior to the last, for medicinal use. Leaves sensitive.

418. C. nictitans. Winking cassia. Wild sensitive plant. Ju.–July. *y.* 1 f. ☉. Sandy places. rb. Mar. O. Ky.
419. C. tora. Jul. *y.* 3 f. ☉. Ky. *St.*
420. C. aspera. Rough cassia. ☉. 1 to 3 f. Ky. *St.*
421. Gymnocladus canadensis. Coffee bean. Coffee tree. May–June. *w.* 30 to 50 f. ♄. O. Ky.
422. Gleditschia triacantha. Honey-locust. June. *w* or *y–w.* 20 to 60 f. ♄. Woods. W.
423. Cercis canadensis. Judas tree. Red bud. Ap. *r.* 10 to 30 f. ♄. Woods. W.

### LXXVIII. *URTICEÆ.*—The nettle tribe.

424. Urtica canaduəsis. Canadian nettle. July–Aug. *g–w.* 3 to 6 f. ♃. sw. Damp. W.

425. U. divaricata, *Beck.* July–Aug. 2 to 3 f. ♃. rs. sw. O?

426. U. pumila. Richweed. Stingless nettle. Jul. 8 to 20 i. ☉. bt. sw. O. Ky.

427. U. diocia. Common nettle. Large stinging nettle. Ju.–Aug. *g.* 2 to 3 f. ♃. Waste places. rd. O. Ky.

428. U. procera, *Muhl.* Tall nettle. Gregareous nettle. Jul.–Aug. *g.* 3 to 4 f. ♃. O?

429. U. gracilis, *Lin.* Slender stalked nettle. June–Aug. *g.* 2 to 3 f. ♃. rs. &c. Hill two miles E. from Cin.

430. U. urens. Dwarf stinger. Stinging nettle. June–July. 12 to 20 i. ☉. cf. Intr. Ky. *St.*

431. Bœhmeria lateriflora. July. *g.* 2 to 7 f. ♃. sw. O. Ky.

432. B. cylindrica. False nettle. June–Aug. *g.* 2 to 3 f. ♃. wp. &c. Two miles S. from Columbus, O.

433. Parietaria pennsylvanica. Pellitory. June. 12 to 15 i. ☉. rs. ow. &c. W.

434. Cannabis sativa. Hemp. June. *w–g.* 6 to 8 f. ☉. Intr.

435. Humulus lupulus. Common hop. Hop vine. Aug. *g–w.* Twining. ♃. bt. Hedges. W. Tonic, narcotic. Big. Med. Bot. iii. 163.

### LXXIX. *ULMACEÆ.*—The elm tribe.

436. Ulmus americana. White elm. Ap.–May. *p.* Tall tree. W. Bark astringent, demulcent. Timber valuable.
437. U. fulva. Slippery elm. May. Large tree. W. Inner bark mucilaginous, demulcent; used considerably in medicine.
438. U. alata. Whahoo. A large tree. Ky. Tenn. *Nutt.*

439. Celtis crassifolia. Hackberry. May. *g–w*. Tree 20 to 40 f. O. Ky.

440. C. occidentalis. Hoop ash. Beaver wood. Nettle tree. May. *g–w*. Tree of middling size. Five miles west from St. L. *Bk.*

441. C. pumila. May. *g*. 6 f. ♄. Banks of rivers, Ky. *St.*

442. Planera aquatica. May. ♄. 25 to 30 f. West of the Alleghanies, *Nutt.*

LXXX. *ARTOCARPEÆ.*—The bread-fruit tribe.

443. Maclura aurantiacia. Maclure's mock orange. Ossage apple. Bow-wood. Yellow-wood. Tree 20 to 30 f. Ark. *Jas.*

444. Morus rubra. Red mulberry. May. 15 to 40 f. ♄. Woods. Ky. O. Fruit very similar to blackberries, edible.

LXXXII. *CUPULIFERÆ.*—The oak tribe.

Plants of this order possess more or less astringency.

445. Quercus imbricaria. Shingle oak. Laurel-leaved oak. Tree 40 to 50 f. Leaves entire. O. Ky. &c. Much used for making shingles, where pine cannot be obtained.

446. Q. discolor. False red oak. May. Large tree 60 to 70 f. Forests, Mar. O.

447. Q. palustris. Pin oak. Water oak? May. Tree 40 to 60 f. bt. Dry woods. Swampy woods. W. Wood firm and useful.

448. Q. obtusiloba. Iron oak. Burr oak, (in Ohio.) May. 20 to 50 f. Sterile grounds, Darby Plains, O. Ky. Timber inferior only to live oak, Q. virens.

449. Q. macrocarpa. Over-cup oak. May. Large tree 40 to 60 f. Limestone hills, *Bk.* bt. Acorn very large. Timber excellent. W.

450. Q. olivæformis. Mossy-cup oak. May. Large tree, much like the preceding. bt. &c. Miami country.

451. Q. alba. White oak. May. A tree sometimes of gigantic dimensions. Forests, where the soil is productive. W. Bark astringent, antiseptic, tonic. Big. Med. Bot. Timber valuable.

452. Q. montana. Rock chesnut oak. Mountain oak. May 30 to 40 f. ♄. rs. O. Excellent fuel. Bark esteemed by tanners.

453. Q. phellos. Willow oak. May. 30 to 60 f. ♄. Swampy forests. Ky. *St.* Ark. *Jas.* Timber of little use.

454. Q. tinctoria. Quercitron. Black oak. May. 70 to 80 f. ♄. Woods. W. Bark used in dying yellow. Astringent, tonic, &c. Disp. Wood and Bache, 524. Michx. N. A. Sylv. i.91.
455. Q. ferruginea, *Michx.* Black Jack. Barren oak. May. 20 to 30 f. ♄. Sandy woods. Kentucky barrens, *St.* Esteemed for fuel. Q. nigra, *Willd.*
456. Q. falcata. Spanish oak. Red oak. May. 70 to 80 f. ♄. Sandy soil. Ky. *St.* Bark esteemed by tanners; wood used for staves and fuel.
457. Q. castanea. Yellow oak. May. 60 to 70 f. ♄. Mountains, &c. Ky. *St.* O. Bark used for dying yellow.
458. Q. pumila. Dwarf oak. Ap. 2 f. ♄. Ky. *St.*
459. Q. chinquapin. Chinquapin oak. Dwarf chesnut oak. May. 3 to 4 f. ♄. Barrens. Brushy Prairie near Dayton, O.
460. Q. bannisteri, *Michx.* Scrub or shrub oak. May. 4 to 6 f. ♄. Barrens. Brushy Prairie?
461. Q. triloba. Downy black oak. May. 20 to 70 f. ♄. Barrens. Brushy Prairie.
462. Q. coccinea. Scarlet oak. May. 70 to 80 f. ♄. Fertile woods. Louisville, *M'Murt.* Used for staves and fuel.
463. Q. cinerea. Ap. 20 f. ♄. Louisville, *M'Murt.*
464. Castanea vesca. Chesnut tree. May–June. Large tree. W.
465. C. pumila. Chinquapin. May. 6 to 12 f. Ky. Mar. O. Nut small and sweet.
466. Corylus americana. Hazel-nut. Wild filbert. March–Ap. 4 to 8 f. ♄. sw. Barrens. W.
467. Fagus ferruginea. Red beech. May–June. Large tree. Woods. O.
468. F. sylvatica. White beech. May. Large tree. Ky. *St.*

LXXXIII. *BETULINEÆ.*—The birch tribe.

469. Betula rubra. Red birch. May. 60 f. ♄. Banks of streams. The summer of the great flood of 1832, I saw a single prostrate but living trunk of the red birch, partly covered by alluvion, on the shore of an island in the Ohio near Marietta. It might have come from the highlands of Pennsylvania or Virginia.
470. B. glandulosa. Scrub birch. May. 2 f. ♄. Mountains. N. States to N.W. Ter. *Bk.*
471. Alnus serrulata. Alder. March. 6 to 10 f. ♄. mh. Swamps. bt. Ky. *St.*
472. A. glauca. March. ♄. St. Croix riv. *Nutt.* N.W. Ter. *Houghton.*

473. Carpinus americana. Blue beech. Water beech. Hornbeam. May. *g–y.* 10 to 20 f. ♄. Damp woods, &c. W.
474. Ostrya virginica. Iron wood. Hop hornbeam. May. 20 to 40 f. ♄. Woods. W.

LXXXIV. *SALICINÆ.*—The willow tribe.

475. Salix myricoides. Ap. ♄. Small shrub. md. rb. Woods, *Bk.* Mar. O. Mich. *Eat.*
476. S. purshiana, *Spreng.* S. falcata, *Pursh.* Pursh's willow. Ap.–May. 8 to 15 f. ♄. bt. Banks of streams, O.
477. S. angustata, *Pursh.* Ap. ♄. A shrub. W. *Muhl.*
478. S. longifolia. Long-leaf willow. Jul. 2 f. ♄. Banks of rivers. W. *Muhl.*
479. S. muhlenbergiana. Speckled willow. Ap. 3 to 5 f. ♄. Dry woods. Mich. *Ect.*
480. S. rosmarinifolia. Rosemary willow. March. 1 to 3 f. ♄. md. wp. Mich. *Eat.* O.
481. S. conifera. Rose willow. Cone-gall willow. Ap. 4 to 8 f. sw. *Bk.* wp? Mich. *Eat. O.*
482. S. ambigua. Ap. ♄. Low grounds, Germantown, O. *Frank.*
483. S. recurvata. Shrub willow. Ap. Low shrub. 2 to 3 f. sw. Germantown, *Frank.*
484. S. tristis. Mourning willow. Ap. 3 to 4 f. ♄. Germantown, *Frank.*
485. S. nigra. Black willow. Brittle-joint willow. Ap.–May. 15 to 20 f. ♄. Banks of streams, Mich. *Eat.* This and other species are tonic, astringent, and antiseptic.
486. S. lucida. Shining willow. May. Small tree or large shrub. Low grounds. Mich. *Eat.*
487. S. grisea. Gray willow. Ap. 3 to 8 f. ♄. Low grounds, Louisville, *M'Murt.*
488. S. babylonica. Weeping willow. May. Ornamental tree. Intr.
489. Populus tremuloides. Quaking asp. Aspen tree. Ap. 20 to 30 f. ♄. Woods, Ky. *St.* Wor. O.
490. P. angulata. Water poplar. Ap. 80 f. ♄. Western rivers, *Jas.*
491. P. lævigata. Cotton wood. Mar. 70 to 80 f. ♄. bt. rs. W.
492. P. candicans. Balm of Gilead. Mar. 40 to 50 f. ♄. Woods, Ky. *St.* Mich. *Eat.* The viscid buds are used in the composition of healing salves.
493. P. heterophy lla. Various-leaved poplar. May. 60to 80 f. Swamps. wp. Darby Plains, O. W. *Bk.*

357

494. P. grandidentata. Large American aspen. Ap. 40 to 50 f. Hills, near Cin. O.

LXXXV. *PLATANEÆ.*—The plane tribe.

495. Platanus occidentalis. Sycamore. Buttonwood. American plane tree. May. 60 to 100 f. ♄. bt. W.
496. Liquidambar styraciflua. Sweet gum tree. May. Large tree. Low woods. W. Fragrant, aromatic. Said to be medicinal.

LXXXVI. *MYRICEÆ.*—The gale tribe.

497. Myrica cerifera. Bayberry. Wax myrtle. May–June. 2 to 10 f. ♄. Shady woods. Sandy situations, &c. Presque Isle, Lake Erie. The berries yield a valuable greenish wax. Astringent, emetic, narcotic. Beach. Big. Med. Bot. iii.
498. M. gale. Sweet gale. Dutch myrtle. May. 3 to 5 f. ♄. bg. Lake Sup. *Houghton.*
499. Comptonia asplenifolia. Sweet fern. Ap.–May. 2 to 4 f. ♄. Woods, &c. Louisville, *M'Murt.* Astringent, tonic. Bart. Med. Fl. i. 224. Raf. Med. Bot. i. 115.

LXXXVII. *JUGLANDEÆ.*—The walnut tribe.

500. Juglans nigra. Black walnut. Ap.–May. 50 to 60 f. ♄. Fertile woods. W. A most valuable and beautiful timber, resembling mahogany.
501. J. cinerea. Butter-nut. White walnut. April–May. Large tree. bt. &c. W. Cathartic. Big. Med. Bot. i. 115.
502. Carya alba. Shag-bark hickory. Ap.–May. Large tree. Fertile woods. W. Timber valuable.
503. C. sulcata. Shell-bark hickory. Ap.–May. Large tree, excellent for fuel, &c. Fertile soils, Ky. O.
504. C. amara. Bitter-nut. May. Large tree. Woods. O. Ky.
505. C. porcina. Pig-nut. Broom hickory. May. Large tree, with tough wood. Woods. Ky. *St.*
506. C. tomentosa. Common hickory. Ap.–May. Large and valuable timber tree. Fertile woods. W. O.
507. C. olivæformis. Pecan nut. Ap. Grows in West. Tenn. Ky. *St.* Louisville, *M'Murt.*

LXXXVIII. *EUPHORBIACEÆ.*—The euphorbium tribe.

508. Euphorbia corollata. Flowering spurge. July–Aug. *w.* 1 to 2 f. ♃. dp. cf. O. Ky. Emetic, diaphoretic, expectorant. Big. Am. Med. Bot. iii. 119. Wood and Bache, Disp. 288.

509. E. obtusata. Jul.–Aug. 12 to 18i. ♃. rd. &c. Shores of Lake Erie.

510. E. peplus. Jul.–Aug. 6 to 18i. ⊙. cr. rd. cf. O. Ky.

511. E. maculata. Spotted spurge. French purslane. Aug.–Oct. generally prone, or creeping. 6 to 12i. ⊙. cf. rd. W.

*b.* var. parvula, *mihi.* Dwarf spotted spurge. Jul.–Oct. Creeping, 3 to 8i. ⊙. rd. O. Leaves scarce one tenth the size of the preceding.

512. E. hypericifolia. Aug.–Sept. Erect, 1 to 2 f. ⊙ rd. Ky. *St.*

513. E. polygonifolia. Knot-grass-leaved spurge. Jul.–Sept. Procumbent, 8i. ♃. Sands, Ky. *St.* Banks of the Ohio and Miss. *Pursh.*

514. E. thymifolia. Ky. *St.* Borders of the Ohio, *Michx.*

515. E. dentata. Toothed-leaf spurge. Jul.–Aug. ⊙. Tenn. *Michx.* Jul. ⊙. Plt. *Jas.*

516. E. portulaccoides. Purslane spurge. June–Aug. ♃. Mo. Ter. *Jas.*

517. E. mercurialina. Jul. ♃. rs. Ky. *Pursh.*

518. E. ipecacuanha. American ipecacuanha. June. ♃. Sandy soil, Louisville, (?) *M'Murt.* Emetic, diaphoretic. Bart. Med. Bot. i. 218. Big. Med. Bot. iii. 109.

519. E. pilosa. Hairy spurge. June–Jul. 1 to 3f. ♃. Wet woods, Wheeling? *Colby.*

520. E. herronii, *mihi.* Herron's euphorbium. *Root* small, branching and fibrous: *stem* erect, pilose: *branches* decussately opposite: *petioles* ciliate, half as long as the leaves: *leaves* varying from the lowest on the stem which are rhomb-ovate, to those of the involucre, which are lance linear; dentate-serrulate, obtuse, at the base acute and entire; pilose above, veins and midrib very hairy beneath: *umbels* small, terminal, involucred and few flowered. Aug.–Sept. ⊙. 1 to 2f. Small prairie half a mile south of Middletown, Ohio. Dedicated to my friend Dr. O. M. Herron, a devoted student in natural science.

521. Croton capitatum. ⊙. Ky. *St.*

522. C. ellipticum. ⊙. St. L. *Nutt.*

523. Crotonopsis linearis. June. 12 to 18i. ⊙. Sandy swamps. W. *Bk.*

524. Acalypha virginica. Three-seeded mercury. June–Aug. 12 to 18i. ⊙. rd. ow. O. Ky. Expectorant, diuretic, *Dr. Atkins.* Elliott's Bot. ii. 645.

525. A. caroliniana, *Walter*. Beck's Bot. 311. Jul.–Aug 9 to 18i. ⊙. ow. &c. O.

526. A. ostryafolia, *mihi*. Iron-wood-leaved mercury. It is probably the A. caroliniana of Elliott, vol. ii. 645. Found by Mr. T. G. Lea, on one of the Kentucky hills opposite Cincinnati. Aug.–Sept. ⊙? The leaves resemble in form, those of Ostrya virginica.

527. Pachysandra procumbens. June. *g--w*. Procumbent. ♃. Ky. *St.*

528. Tragia macrocarpa. Jul. ⊙. Climbing. Ky. *Michx.*

XCIII. *CELASTRINEÆ.*—The false bitter-sweet tribe.

529. Celastrus scandens. False bitter-sweet. Red root. Climbing staff tree. May–June. *g.--y*. Woody vine, 10 to 20f. Woods. Narcotic, secernant stimulant. It is regarded by Mr. Miles, a Thompsonian, as stimulating and diuretic, and capable of removing hepatic obstructions. Portio pulv. cort. radicis, ℥i. ter die.

530. Euonymus atropurpureus. Indian arrow. June. *p*. 6 to 10f. ♄. sw. O. Has active medicinal virtues, if we credit popular opinion.

531. E. americanus. Burning bush. Spindle tree. June. *r--y*. 4 to 6f. ♄. sw. Pittsburgh, *Peter*. Ky. *St.*

532. E. obovatus. Trailing euonymus. June. *g*. & *p*. Prostrate, 2 to 6f. long. sr. Ky. Wor. O. Root similar to that of sp. 529, and probably possesses similar qualities.

XCVI. *RHAMNEÆ.*—The buckthorn tribe.

533. Rhamnus carolinianus. May. 4 to 6f. Ky. *St.*

534. R. alnifolius. Dwarf elder. May–June. *g*. ♄. rs. Mo. Ter. *Eat.*

535. R. lanceolatus. Shrub. Ky. *St.*

536. Ceanothus americanus. Red-root. New Jersey tea. Jul. *w*.2 to 3f. ♄. ow. dp. W. An Indian remedy for syphilis. Hoop. Med. Dic. Astringent. Raf. Med. Bot. ii. 205.

537. C. sanguineus. Bloody ceanothus. May. *w*. 3 f. ♄. Plt. *Nutt.*

538. C. intermedius, *Pursh*. Dwarf red-root. Similar to 536, but much smaller. *Elliott*. Mo. Ter. *Jas.* Woods, Tenn. *Lyon*. Pursh, i. 167.

XCVII. *STAPHYLEACAÆ.*—The bladder-nut tribe.

539. Staphylea trifolia. Bladder-nut. Ap.–June. *w*. 6 to 10f. ♄. sw. sr. cr. W.

XCVIII. *HIPPOCASTANEÆ.*—The horse-chesnut tribe.

540. Æsculus glabra, *Willd.* May. *y—w.* 10 to 30 f. ♄. Woods. Lexington, *Short*, Eat. Man. Banks of the Illinois, *Bk.* Probably identical with the next.

541. Æ. ohioensis, *mihi.* A. pallida, *Willd.* ohioensis, *Eat? Michx?* Buckeye. Fetid buckeye. *Calyx* half as long as the corol: *petals* 4, yellowish, claw shorter than the calyx: *stamens* twice as long as the petals: *pericarp* echinate: *leaves* in fives, lance-oblong, acuminate, serrulate, glabrous above, axils of the veins pubescent beneath: *bark* rough, cinereous. 40 to 50 f. ♄. bt. O. Ky. Emetic, narcotic, &c. *Dr. Drake.*

542. Æ. flava, *Ell.* Bot. i. 436. *Pursh.* Æ. lutea, *Linn? Eat?* Sweet buckeye. Upland buckeye. Ap. Pale *y.* 20 to 80 f. ♄. Pericarp smooth. Hills near large rivers, O. Ky.

543. Æ. On the Walnut Hills near Cincinnati, I find a species of Æsculus resembling the flava, but differing in the deep orange and yellow hue of its flowers, its glabrous, irregularly serrate leaves, and more acute divisions of the calyx. The buckeye (either the flava or ohioensis) is regarded as the emblem tree of Ohio.

544. Æ. discolor, *Pursh.* May. *y. w.* & *p.* 4 f. ♄. West Va. *Paddock.*

C. *SAPINDACEÆ.*—The soap-tree tribe.

545. Cardiospermum halcacabum, *Willd.* Heart seed. Aug. ⊙. Mo. Ter. *Jas.* Aperient, Lind. Nat. Syst. 115.

CI. *ACERINEÆ.*—The maple tribe.

546. Acer rubrum, *Ehrh.* Red maple. Soft maple. Ap. *r.* 30 to 50 f. ♄. Woods. W. Bark astringent: used domestically in dying black.

547. A. eriocarpum, *Michx.* White maple. Silver maple. River maple. Ap.–May. *g.* Large tree. River banks. W.

548. A. saccharinum, *Linn.* Sugar maple. Hard maple. Ap.–May. *g–y.* 40 to 60 f. Woods W. Yields sugar and excellent fuel.

549. A. nigrum, *Michx.* Black maple. Sweet tree. Ap. *y–g.* 40 to 60 f. Very similar to the last. O.

550. A. negundo, *Linn.* Ash-leaved maple. Box elder. Ap. *g.* 30 to 60 f. ♄. bt. & W.

551. A. striatum, *Michx.* Striped maple. False dogwood. May. *g.* 15 f. Woods. Louisville, *M'Murt.*—

For the medicinal virtues of the maples, see Raf. Med. Bot. ii. 185.

CIV. *AMPELIDEÆ*, *De Cand.*—The vine tribe.

552. Vitis vulpina, *Linn.* Winter grape. Frost grape. June. *w–g.* Woody vine. 5 to 50 f. River banks, bt. O. Ky. Fruit tart, Nov.

553. V. æstivalis, *Michx.* Summer grape. May–June. *w–g.* Woody vine, 5 to 50 f. sw. bt. Missouri banks, St. L. *Bk.* O. Fruit, Aug.

554. V. labrusca, *Linn.* Fox grape. Longworth's grape. June–July. *w–g.* Woody vine, 10 to 60 f. Woods, O. Ky. Cultivated in Ohio.

555. V. riparia, *Michx.* Fragrant flowered grape. July. *w–p.* Woody vine. Gravelly shores, Mich. *Eat.*

556. Ampelopsis quinquefolia, *Michx.* Common creeper. American ivy. June–Jul. *g.* Woody vine, 5 to 40 f. ow. rs. W. Admirable expectorant in pulmonary complaints. The vine. Mr. Lane of Norwalk, Ohio. Eberle, West. Med. Gaz. i.

557. A. cordata, *Michx.* Heart-leaved ivy. Ju.–Jul. Woody vine. Banks of streams. Alleghany Mountains, *Eat.* West to Ark. *Bk.*

558. A. bipinnata, *Michx.* June–July. Shrubby vine. Rich, damp soils. Ark. *Jas.*

CXIII. *ANACARDIACEÆ.*—The cashew tribe.

559. Rhus glabra, *Linn.* Sumach. Smooth sumach. July. *g–y.* 6 to 12 f. ♄. Old fields, &c. W. Berries astringent, refrigerant. Am. Jour. Med. Sci. v. 61. Big. Med. Bot.

560. R. copallina, *Linn.* Mountain sumach. July. *y–g.* 3 to 12 f. ♄. Dry fields, Ky. *St.* Yields gum copal.

561. R. aromatica. Aromatic sumach. May–June. *y.* 2 to 6 f. ♄. bt. Calcareous regions. W. Medicinal?

562. R. vernix, *Linn.* Poison ash. June. *g–y.* 10 to 15 f. ♄. Margins of swamps. O. Stimulant, poisonous. Big. Med. Bot. i. 96.

563. R. toxicodendron, *Linn.* Poison vine. Poison ivy. June. *w–g.* 2 to 5 f. ♄. Woods. W. Stimulant, narcotic. Big. Med. Bot. iii. 19. Juice extremely poisonous.

*b.* var. radicans, *Torr.* Climbing trees and rooting in their bark. 30 to 50 f. Supposed by some to cause the milk sickness.

564. R. typhina, *Linn.* Stag's horn. June. *g–y.* 10 to 15 f. rs. Louisville, *M'Murt.*

CXIV. *XANTHOXYLEÆ.*—THE PRICKLY ASH TRIBE.

565. Xanthoxylum fraxineum, *Willd.* Prickly ash. Ap. *g.* 3 to 5 f. ♄. Woods, W. Stimulant, diaphoretic, sialagogue, antirheumatic. Big. Med. Bot. iii. 156.
566. Ptelea trifoliata, *Linn.* Stinking prairie bush. Stinking ash. Swamp dogwood. Shrubby trefoil. June. *g–w.* 4 to 10 f. ♄. ow. dp. W. Said to cure intermittents. Doubtless actively medicinal.

CXXII. *GERANIACEÆ.*—THE GERANIUM TRIBE.

567. Geranium maculatum, *Linn.* Cranesbill. June. *p.* 8 to 15 i. ♃. Woods. Root astringent. Big. Med. Bot. i. 19.
568. G. carolinianum, *Linn.* Jul. *w* & *r.* 12 to 18 i. ♂. wp. Ill. riv. *Bk.*

CXXIII. *OXALIDEÆ.*—THE WOOD-SORREL TRIBE.

The western plants of this order are acidulous and refrigerant, and may be used in fevers. Said to be antiscorbutic. Wood & Bache. 10.

569. Oxalis stricta, *Linn.* Wood-sorrel. May–Aug. *y.* 4 to 10 i. ⊙. Sandy fields, &c. O. Ky.

570. O. violacea, *Linn.* Violet wood-sorrel. May–June. *p.* 4 to 6 i. ♃. Hills. rs. W.

571. O. corniculata, *Linn.* May–Aug. *y.* 6 to 10 i. ⊙. Woods, sr. &c. O.

572. O. recurva, *Ell.* Ap.–May. *y.* 4 to 8 i. ⊙. cf. rb. Mar. O.

573. O. dillenii, *Willd.* May. *y* & *p.* 6 to 18 i. ⊙. Woods, O.

CXXVI. *BALSAMINEÆ.*—THE BALSAM TRIBE.

574. Impatiens pallida, *Nutt.* Snap weed. Touch me not. False celandine. Aug. pale *y.* 2 to 4 f. ⊙. Damp grounds. W. Diuretic, emetic. Raf. Med. Bot. ii. 231. Lind, 140.
575. I. fulva, *Nutt.* Jewel weed. Aug. *y* & *orange.* 2 to 3 f. ⊙. Damp grounds, W. Medicinal, like the last.

CXXIX. *POLYGALEÆ.*—THE MILKWORT TRIBE.

576. Polygala senega, *Linn.* Seneka snakeroot. June–Jul. *w.* 1 f. ♃. Woods. O. Ky. Stimulant, diuretic, sialagogue, expectorant, purgative, emetic, sudorific, emenagogue! Bart. ii. 116. Big. ii. 97.
577. P. ambigua, *Nutt.* Jul. *p.* 8 to 10 i. Mar. O.
578. P. alba, *Nutt.* *w.* 6 i. ♃. Plains, Mo. Ter. *Nutt.*

579. P. polygama, *Walt.* Bitter polygala. June–July. *p.* 4 to 8 i. ♃. Woods. Mich. *Nutt.* Medicinal, bitter. Big. Med. Bot. iii. 129.
580. P. brevifolia, *Nutt.* Jul.–Aug. *r.* ⊙. Sandy swamps, O. *Nutt.*
581. P. purpurea, *Nutt.* Jul.–Aug. *p–r.* 12 to 18 i. ⊙. Duncan's Plains, O.
582. P. incarnata, *Linn.* Milkwort. June–Jul. *r.–p.* 12 to 18 i. ⊙. Mich. *Eat.* Ky. *St.*
583. P. verticillata, *Linn.* Jul.–Oct. *g–w.* 8 to 12 i. ⊙. Sandy soils. W. *Bk.* Ky. *St.*
584. P. setacea, *Michx.* Jul. ⊙. Ky. *St.*
585. P. pauciflora, *Willd.* Flowering wintergreen. June. *p.* 3 to 4 i. ♃. Woods, &c. Near Lake Huron, *Bk.*
586. P. sanguinea, *Linn.* Bloody polygala. June–Oct. *r.* 8 to 12 i. ⊙. Dry soils, Louisville, *M'Murt.*
587. P. lutea, *Linn.* Yellow polygala. June–Oct. *y.* 8 to 16 i. ♂. Pine barrens, &c. Louisville, *M'Murt.*

CXXX. *VIOLACEÆ.*—The violet tribe.

Plants of this order possess emetic, cathartic, demulcent and expectorant qualities.

588. Viola cucullata, *Ait.* Common blue violet. May. *b.* 4 to 8 i. ♃. Woods, O.
589. V. pubescens, *Ait.* Yellow wild violet. May. *y.* 6 to 8 i. ♃. Woods, W.
590. V. rostrata, *Muhl.* Ap.–May. Pale *b.* 6 to 8 i. ♃. Woods. rs. &c. Wor. O. Ky.
591. V. affinis, *Le Conte.* Prairie violet. May, *Eat.* Oct. *b–p.* 2 to 6 i. ♃. wp. Van Cleve's Prairie, Dayton. Pickaway Plains, O. Ky.
592. V. palmata, *Linn.* Hand-leaf violet. May. *b–p.* 3 to 6 i. ♃. Cin. O. Ky.
593. V. sororia, *Willd.* May. *b–p.* ♃. Dry woods, Ky. *St.*
594. V. sagittata, *Ait.* Arrow-leaf violet. May. *p.* ♃. Fields. West Va. *Paddock.* Ky. *St.*
595. V. amœna, *Le Conte.* Twisted violet. Ap. *w.* ♃. Moist woods, Ky. *St.*
596. V. blanda, *Willd.* Smooth violet. Ap.–May. *w* & *p.* 2 to 4 i. ♃. Wet meadows, &c. Ky. *St.* West of St. L. *Bk.*
597. V. primulæfolia, *Linn.* Ap.–May. *w.* ♃. Wet woods, Ky. *St.*
598. V. lanceolata, *Linn.* Ap.–May. *w.* 3 to 8 i. ♃. Swamps, Ky. *St.* Near Lake Huron, *Bk.*
599. V. pedata, *Linn.* May. Pale *b.* 3 to 5 i. ♃. rs. Hills,

W. *Bk.* Ky. *St.* Medicinal. Wood & Bache, 668. Curtis Bot. Mag. 89.

600. V. canadensis, *Linn.* Canadian violet. *r, w & b.* 12 to 18 i. ♃. ow. Fields. W. *Bk.* Ky. *St.* Cin. O.

601. V. muhlenbergiana, *De Cand.* Muhlenberg's violet. May. *b.* 6 to 10 i. ♃. *Swamps,* Ky. *St.*

602. V. ochroleuca, *Schw.* V. striata, *Ait.* Striated violet. May. *y–w.* 6 to 10 i. ♃. ow. bt. Swamps, *Bk.* O. Ky.

603. V. heterophylla, *Muhl.* Various-leaved violet. March. *p.* 8 to 12 i. ♃. Ky? *St.*

604. V. debilis, *Michx.* March–Ap. *w.* ♃. Louisville, *M'Murt.*

605. V. nuttalli, *Pursh.* Nuttall's violet. May. *y.* 4 to 6 i. ♃. Banks of the Missouri, *Pursh.*

606. V. tenella, *Muhl.* May. *b–w.* 2 to 4 i. ♃. Sandy Hills, W. *Bk.*

607. V. tricolor, *Linn.* Pansy. Heart's ease. Garden violet. May. *p, y and p–b.* 3 to 8 i. Intr. Medicinal. Wood and Bache, 668.

608. Solea concolor, *De Cand.* Green violet. April–May. *g.* 2 to 4 f. ♃. rs. sw. W. Medicinal?

CXXXI. *PASSIFLOREÆ.*—The passion-flower tribe.

609. Passiflora lutea, *Linn.* Yellow passion-flower. Aug. *y.* Delicate vine, 5 to 10 f. ♃. Thickets, O. Ky.

610. P. incarnata, *Willd.* Flesh-colored passion-flower. May–July. *w & p.* Vine, 20 to 30 f. ♃. Dry soils, Ky. *St.*

CXXXIV. *CISTINEÆ.*—The rock-rose tribe.

611. Lechea villosa, *Ell.* Pin-weed. Jul. Flowers brown. 1 to 2 f. ♃. Barren soil, Ky. *St.*

612. L. minor, *Pursh.* Smaller pin-weed. Jul. *g–p.* 8 to 10 i. ♃. Dry hills, Ky. *St.*

613. L. racemulosa, *Michx.* Jul. 1 to 2 f. ♃. Sandy situations, Mar. O. Ky.

614. Hudsonia tomentosa, *Nutt.* Silvery false heath. June. *y.* 6 i. ♄. Sea shore usually. Mich. *Eat.*

615. H. ericoides, *Linn.* False heath. May–June. *y.* 4 to 6 i. ♄. Pine barrens, *Bk.* Prairie near Middletown, O (?) Louisville, *M'Murt.*

616. Helianthemum canadense, *Michx.* Rock-rose. Frost weed. June. *y.* 1 f. ow, &c. Sandy prairie near St. L. *Bk.*

CXXXVI. *SARRACENIEÆ*

617. Sarracenia purpurea, *Linn.* Side-saddle flower. June–

July. *p.* 1 to 2 f. ♃. bg. Said to occur in the northern part of Ohio, Doubtful.

CXXXVII. *DROSERACEÆ.*—THE SUNDEW TRIBE.

618. Drosera longifolia, *Linn.* Long-leaved drosera. July–Aug. 2 to 4 i. ♃. Swamps. Near Lake Sup. *Hough.* Ky. *St.*
619. D. rotundifolia, *Linn.* Sundew. July. *w.* 4 to 8 i. ♃. Swamps. Ky. *St.* Lk. Sup. *Hough.* Acid, slightly acrid. Lind. 151.

CXXXIX. *LINEÆ*—THE FLAX TRIBE.

620. Linum virginanum, *Linn.* Wild flax. July. *y.* 1 to 2 f. ⊙. rs. O. Ky.

621. L. perenne, *Linn.* Perennial flax. Jul. *b.* 1 to 2 f. ♃. Mo. Ter. *Nutt.*

622. L. rigidum, *Linn.* *y.* 6 i. Mo. Riv. *Jas.*

CXL. *CARYOPHYLLEÆ.*—THE CHICKWEED TRIBE.

§ 1. SILENEÆ. *Pink like.*

623. Silene virginica, *Linn.* Virginian catch-fly. May–June. *r.* (*purple* according to Beck.) 1½ to 3 f. ♃. ow. bt. O. Anthelmintic. Lind. Nat. Syst. 154.
624. S. pennsylvanica, *Michx.* Wild pink. May–June. *r.* 8 to 12 i. ♃. Sandy woods. Mar. O. Ky. Mich.
625. S. regia, *Linn.* Royal catch-fly. Jul. *r.* 2 to 3 f. ♃. Prairies, O, Ky.
626. S. catesbæi, *Walt.* June. *Crimson.* 1 f. ♃. Penn. to Miss. *Muhl.*
627. S. rotundifolia, *Nutt.* Round-leaved catch-fly. Jul. *r.* rs. O. and Tenn. *Nutt.* Ky. *St.*
628. S. acaulis, *Linn.* Jul. *r.* ♃. White Mountsins, N. H. west to the Rocky Mountains, *Bk.*
629. Agrostemma githago, *Linn.* Corn cockle. June–July. *p.* 1½ to 3 f. ⊙. cf. Intr.
630. Saponaria officinalis, *Linn.* Soap-wort. Bouncing Bet. June–Sept. pale *r.* 12 to 18 i. ♃. rd. rb. Miami country. Intr. Acrid, saponaceous, anti-venereal. Eberle Mat. Med. ii. 220.
631. Cucubalus stellatus, *Linn.* Star campion. Jul. *w.* 2 to 3 f. ♃. cr. ow. O. Ky. Prairies, Ill. and Mo. *Bk.*

§ 2. ALSINEÆ. *Chick-weed like.*

632. Sagina procumbens, *Linn.* Pearl-wort. Jul. *w.* 2 to 4 i. ♃. Borders of streams, W. *Bk.*

633. Spergula arvensis, *Linn.* Corn spurrey. June–Aug. *w.* 6 to 12 i. ☉. Sandy fields. Intr. W. *Bk.*

634. S. saginoides, *Linn.* Pearl-wort spurrey. June. *w.* 2 to 4 i. ☉. Sandy fields. W. *Bk.* Very similar in appearance to sp. 632.

635. Mollugo verticillata, *Linn.* Carpet weed. Indian chickweed. July–Sept. *w.* Prostrate. cf. Sandy situations, W.

636. Stellaria media, *Smith.* Chickweed. March–Nov. *w.* Spreading, 3 to 6 i. ☉. rd. W.

637. S. pubera, *Michx.* Starwort. May. *w.* 6 to 10 i. ♃? rs. W.

638. S. palustris, *Retz.* Stitchwort. June. *w.* 6 to 12 i. mh. Wor. O.

639. S. uliginosa, *Screb.* Bog starwort. June. *w.* (4 to 6 i. ☉? Woods, cr. O)?

640. S. longifolia, *Muhl.* June. *w.* 12 to 15 i. ♃. Moist woods, St. L. *Bk.*

641. Cerastium nutans, *Raf.* June. *w.* 6 to 12 i. ☉. rs. Hills, *Bk.* Banks of rivulets, Wor. O. Ky. Mich.

642. C. vulgatum, *Linn.* Mouse-ear chickweed. May–Aug. *w.* 6 to 10 i. ☉. Fields and hills. Intr. W.

643. C. viscosum, *Linn.* Sticky chickweed. May–Aug. *w.* 6 to 12 i. ♃. rd. cf. Mich. *Eat.*

644. C. hirsutum, *Muhl.* Hairy chickweed. May. *w.* 4 to 8 i. ☉. Dry hills, Ky. *St.*

645. C. arvense, *Willd.* June. *w.* 6 i. ♃. Ky. *St.*

646. C. dichotomum, *Muhl.* June. *w.* 9 i. ☉. Pittsburgh. *Peter.*

647. C. oblongifolium, *Torr.* June. *w.* 8 to 10 i. ♃. Mountains, *Bk.* Mich. *Houghton.*

648. Arenaria stricta, *Michx.* Erect sandwort. May–June. *w.* 6 to 12 i. ♃. Mountains, *Bk.* Mich. *Eat.* Ky. *St.*

649. A. juniperina, *Willd.* Juniper leaved sandwort. ♃. Mich. *Eat.*

650. A. serpyllifolia, *Linn.* Thyme leaved sandwort. May–Jul. *w.* Decumbent, 3 to 8 i. ☉. Sandy fields, Ky. *St.*

651. A. lateriflora, *Linn.* Sandwort. June. *w.* 5 to 10 i. ♃. md. Wor. O. Lk. Sup. *Hought.*

652. A. obtusa, *Torr.* ♃. Mich. Eat.

653. A. patula, *Michx.* ♃. Knoxville, Ky. *Michx.*

### CXLIII. *ELATINEÆ.*—The water-pepper tribe.

654. Crypta minima, *Nutt.* Mud purslane. May–Aug. *g–w.*

Prostrate, small. ⊙? Banks of streams, throughout U. S. *Bk.*

CXLIV. *PORTULACEÆ.*—THE PURSLANE TRIBE.

655. Portulacca oleracea, *Linn.* Purslane. May–Aug. *y.* Spreading, 6 to 18 i. ⊙. cf. Intr. W. Indigenous to the plains of Mo. *Nutt.* Used as a potherb.
656. Claytonia virginica, *Linn.* Spring beauty. Ap. *w* & *r.* 6 to 12 i. ♃. ow. md. W.
657. C. lanceolata, *Pursh.* June. *w.* ♃. Louisville, *M'Murt.*
658. Talinum teretifolium, *Pursh.* Taliny. Jul.–Sept. *p.* 4 to 10 i. ♃. rs. St. L. *Bk.* St. Croix riv. *Houghton.*

CXLVI. *GALACINEÆ.*

659. Galax rotundifolia, *Michx.* May–Aug. *w.* 12 to 18 i. ♃. Mts. Tenn. *Nutt.*

CXLVII. *CRASSULACEÆ.*—THE HOUSE-LEEK TRIBE.

Possess refrigerant and abstergent properties. Lind. Nat. Syst. 160.

660. Sedum ternatum, *Michx.* False ice-plant. June. *w.* 2 to 3 i. ♃. rs. cr. W.

661. S. pulchellum, *Michx. p.* 3 to 4 i. Ky. *St.*

662. Penthorum sedoides, *Linn.* Virginian stone crop. Virginian orpine. Jul.–Aug. Pale *y.* 12 to 18 i. ♃. rb. bt. W.

CL. *ILLECEBREÆ.*

663. Queria canadensis, *Linn.* Forked chickweed. Jul.–Aug. *w.* 6 to 12 i. ⊙. ow. sw. O. Ky.
664. Q. argyrocoma, *Muhl.* June. rs. Tenn. *Nutt.*
665. Q. sessiliflora, *Nutt.* Jul. Hills Mo. Ter. *Nutt.*

CLI. *AMARANTACEÆ.*—THE AMARANTH TRIBE.

666. Amarantus albus, *Willd.* White coxcomb. Jul. *g–w.* 18 i. ⊙. Gardens, cf. O.
667. A. hybridus, *Willd.* Jul. *g.* 1 to 2 f. ⊙. cf. rd. O.
668. A. græcizans, *Willd.* Aug. *g.* ⊙. Louisville, *M'Murt.*
669. A. altissimus, *mihi.* Tall amaranth. *Spikes* lax, simple when axillary, compound when terminal: *leaves* ovate-oblong, mucronate, acute at the base, long petioled: *stem* branching above, 6 to 8 f. Flowers reddish green. Aug. ⊙. On an old prairie, near Hamilton, O.
670. A. miamiensis, *mihi.* Prairie amaranth. *Spikes* rather lax, axillary and terminal, compound: *leaves* ovate and lance-ovate, retuse, mucronate, acute at the base: *pe-*

*tioles* nearly as long as the leaves. Very branching, 2 to 3 f. ☉. Flowers *r* & *g*. Hoffman's prairie, near Dayton, O. I give these as merely temporary names, until the plants shall be further investigated.

671. Iresine celosioides, *Willd.* Sept.–Oct. *w.* 3 to 4 f. ☉. rb. &c. Cin. O. Ky.

CLIII. *CHENOPODEÆ.*—The goosefoot tribe.

672. Chenopodium album, *Linn.* Lamb's quarter. Pigweed. Jul.–Aug. *g.* 2 f. ☉. cf. rd. W. A potherb.
673. C. rhombifolium, *Muhl.* June. *g.* 2 to 3 f. cf. rd. Wor. O.
674. C. anthelminticum, *Linn.* Worm-seed. Aug. *g.* 18 to 24 i. ♃ Fields, Ky. O. Anthelmintic. Bart. ii. 187.
675. C. ambrosiodes, *Linn.* Sweet pigweed. Aug.–Sept. *g.* 18 to 24 i. ♃. ☉. rd. O. Tonic, anthelmintic. Lind. 192.
676. C. botrys, *Linn.* Jerusalem oak. Jul.–Sept. *g.* 12 to 16 i. ☉. rd. Ky. O. Pectoral. Hoop. Dic.
677. C. hybridum, *Linn.* Jul.–Aug. 2 to 3 f. ☉. rd. Ky. Intr.
678. C. maritimum, *Linn.* Aug.–Sept. 18 to 24 i. ☉. Salt meadows usually. Plains of the Mo. riv. *Nutt.*
679. Atriplex canescens, *Nutt.* May. 3 to 4 f. Saline hills, Mo. Ter. *Nutt.* Mo. *Bk.*
680. A. argentea, *Nutt.* Silver-leaf orach. 1 f. ☉. Plt. *Jas.*
681. A. patula, *Linn.* Aug. Prostrate, 1 to 2 f. ☉. Louisville, *M'Murt.*
682. A. hortensis, *Linn.* Garden orach. Jul. *g.* 3 to 4 f. ☉. cf. O. Intr. A potherb.
683. Acnida cannabina, *Linn.* Water hemp. Jul.—Aug. *g.* 3 to 6 f. ☉. mh. Ky. *St.*
684. Corispermum hyssopifolium, *Willd.* Aug. ☉. Plt. *Jas. Nutt.*
685. Blitum capitatum, *Linn.* Strawberry blite. Indian strawberry. June. 15 i. ☉. Fields, &c. N. W. Ter. *Houghton.*
686. B. chenopodioides, *Nutt.* ☉. Dry situations. Plt, *Jas.*
687. B. virgatum, *Linn.* Slender blite. June. ☉. Fields. Intr. Louisville, *M'Murt.*
688. Kochia dioica, *Nutt.* May. 6 to 12 i. ☉. Mo. Ter. *Nutt.*
689. K. dentata, *Willd.* June. ☉. Plt. *Jas.*
690. Diotis lanata, *Pursh.* June. ♄. Arid situations on the banks of the Missouri, *Nutt.*
691. Polycnemum americanum, *Nutt.* ♃. Plt. *Jas.* Mo. Ter. *Nutt.*

CLIV. *PHYTOLACCEÆ.*—Indian poke tribe.

692. Phytolacca decandra, *Linn.* Indian poke. Poke-weed. Scoke-weed. June–Oct. *w.* 4 to 8 f. ♃. ow, &c. W. Emetic, antirheumatic, anti-schirrous? Bart. Med. Fl. ii. 220.

CLVI. *POLYGONEÆ.*—The buck-wheat tribe.

693. Polygonum aviculare, *Linn.* Knot grass. May–Sept. *g.* Procumbent, 6 to 8 i. ⊙, *Bk.* ♃, *Eat.* rd. W. Astringent, styptic. Hoop. Dic.

694. P. erectum, *Muhl* Large knot grass. Aug. *g.* 8 to 30 i. ♃. cf. rd. W. Resembles the preceding.

695. P. virginianum, *Linn.* Virginian bistort. Jul.--Aug. *w* & *r.* 2 to 4 f. ♃. sw. W. Astringent, &c.

696. P. punctatum, *Ell.* Water pepper. Smart weed. Aug.–Sept. *w.* 1 to 2 f. ⊙. rb &c. O. Ky. Acrid, emenagogue, diuretic. Eberle, West. Med. Gaz. i. Hoop. Dic.

697. P. persicaria, *Linn.* Ladies' thumb. Jul.–Aug. *r.* 12 to 18 i. ⊙. Wet grounds, O. Ky. Vulnerary, antiseptic. Hoop. Dic.

698. P. lapathifolium, *Linn.* Aug.–Sept. *w* or pale *r.* 2 to 3 f. ⊙. Ditches, &c. O. This, and the two preceding species, I believe, are used in dying yellow.

699. P. mite, *Pers.* Tasteless knot-weed. Jul.---Sept. *p.* 12 to 18 i. ⊙. mh. pl. Darby plains, O.

700. P. pennsylvanicum, *Linn.* Jul.–Sept. *w–r.* 2 to 4 f. ⊙. Margins of ponds. O. Ky.

701. P. barbatum, *Willd.* Jul. *r–w.* 18 to 24 i. Louisville, *M'Murt.* Seeds carminative. Ainslie, ii. 2.

702. P. bistortoides, *Pursh.* June. *w–r.* ♃. Low grounds on the banks of the Missouri, *Lewis.*

703. P. amphibium, *Linn.* Amphibious knot-weed. Aug. *r.* 12 to 18 i. ♃. pl. mh. O. Ky.

704. P. coccineum, *Willd.* Creeping knot-weed. Aug. *r.* Creeping, 6 to 12 f. *Eat.* ♃. Muddy situations. rb. O.

705. P. hirsutum, *Walt.* Jul. *w.* 2 f. O. (rb. Mar. O.)?

706. P. articulatum, *Linn.* Joint-weed. Sept. *r* & *w.* ⊙. 9 to 15 i. Sandy plains, Mich. *Nutt.*

707. P. sagittatum, *Linn.* Prickly knot-weed. Arrow-leaf knot-weed. July–Aug. *w.* Vine, 4 to 6 f. ⊙. md. wp. W.

708. P. arifolium, *Linn.* Halbert-leaf knot-weed. Aug.–Sept. Pale *r.* 2 to 4 f. ⊙. mh. md. wp. W.

709. P. scandens, *Linn.* Climbing buck-wheat. Jul.–Aug. *w* & *r.* Vine, 4 to 8 f. ⊙. sw. Hedges, W.

710. P. convolvulus, *Linn.* Bind-weed. Jul.–Sept. Reddish *w.* Vine, 6 to 8 f. ☉. cf. Mo. *Bk.* O.
711. P. fagopyrum, *Linn.* Buck-wheat. June. *r–w.* 1 to 2 f. ☉. cf. Ohio banks. Intr.
712. Brunnichia cirrhosa, *Michx.* Ap.–May. Climbing. ♃. Wet places among bushes. Islands, Tenn. *Pursh.*
713. Eriogonum sericeum, *Pursh.* Jul. *y.* ♃. Prairies of Missouri, *Nutt.* (Pursh. i. 277.)
714. Calligonum canescens, *Pursh.* June. ♄. Plains of the Mo. near the Big-bend, *Pursh.*
715. Rumex sanguineus, *Linn.* Bloody dock. June–Jul. 2 f. ♃. rd. Fields. Intr. O. Ky.
716. R. crispus, *Linn.* Curled dock. June–Jul. 2 to 3 f. ♃. Fields. Intr. O. Ky. This and the three following species, are astringent, tonic, alterative and antiscorbutic. Wood & Bache, 545.
717. R. brittanicus, *Linn.* Yellow-rooted water dock. June–Jul. 3 f. ♃. Swamps, W. Medicinal. See 716.
718. R. aquaticus, *Linn.* Water dock. Jul.–Aug. 3 to 4 f. ♃. Ponds and ditches, Wor. O? Med. See 716.
719. R. obtusifolius, *Linn.* Blunt-leaf dock. Jul. 2 to 3 f. ow. Fields. Intr. Mar. O. Med. See 716.
720. R. persicarioides, *Linn.* June–Jul. 6 to 12 i. ☉. sw. Damp places, Ky. *St.*
721. R. acetosella, *Linn.* Sorrel. Sheep sorrel. June–Jul. 6 to 12 i. cf. rd. W. Refrigerant, diuretic, antiscorbutic. Wood & Bache, 9.
722. R. venosus, *Pursh.* Ap. 1 f. ♃. Sandy banks, Plt. *Nutt.*
723. R. crispatulus, *Michx.* Ky. *Michx.*
724. R. verticillatus, *Walt.* June. 2 to 3 f. ♃. (Mh. Swamps, O.)?
725. R. acutus, *Linn.* June. 2 to 3 f. ♃. Moist grounds, Louisville, *M'Murt.* Intr.

CLVIII. *NYCTAGINEÆ.*—The marvel of Peru tribe.

The roots of these plants are more or less purgative. Lind.

726. Alliona abida, *Walt.* Ap. ☉? Mo. Ter. *Nutt.* N. W. Ter. *Houghton.*
727. A. nyctaginea, *Michx.* Jul. ♃. Common on the alluvions of the Missouri. *Nutt.*
728. A. linearis, *Pursh.* Plt. *Jas.*

CLIX. *SAURUREÆ.*—The swamp lily tribe.

729. Saururus cernuus, *Linn.* Swamp lily. Lizard's tail. Breast weed. Aug. *w.* 1 to 2 f. ♃. rb. mh. &c. W. Medicinal?

CLXIII. *PODOSTEMEÆ.*—Thread-foot tribe.

730. Podostemum ceratophyllum, *Michx.* Thread foot. Jul. ♃. Closely attached to loose stones in shallow water of the Ohio and Kentucky rivers. *Pursh.*

CLXV. *CERATOPHYLLÆ.*—The hornwort tribe.

731. Ceratophyllum demersum, *Linn.* Hornwort. Coontail. Jul. Submersed. 1 to 2 f. ♃. pl. O.

732. C. submersum, *Linn.* Jul. Submersed. ♃. pl. Wor. O.

Division 2.—Monopetalous plants.

CLXVI. *ILICINEÆ.*—The holly tribe.

733. Ilex opaca, *Ait.* American holly. June. *w.* 10 to 15 f. ♄. Sandy woods. Ky. *St.*

734. Prinos verticillatus, *Linn.* Winter berry. False alder. June–Jul. *w.* 6 to 8 f. ♄. Swamps, &c. O. The bark and berries are astringent, tonic, antiseptic. Bart. i. 203. Big. iii. 141.

735. P. lævigatus, *Pursh.* Jul. 6 to 8 f. ♄. Swamps. Mo. Ter. *Jas.* W. *Bk.*

736. P. lanceolatus, *Pursh.* June. ♄. (6 to 8 f. Barrens. wp. Duncan's plains, near Columbus, O.)?

CLXIX. *SAPOTEÆ.*—The Sappodilla tribe.

737. Bumelia oblongifolia, *Nutt.* ♄. 18 f. Lead mines near St. Louis. *Nutt.*

CLXX. *ERICEÆ.*—The heath tribe.

§1. *Ericeæ.*—Heath like.

738. Arbutus uva-ursi, *Linn.* Bear-berry. Ap.–May. Pale *r.* Evergreen, trailing vine. ♄. Mts. *Bk.* Sandy situations. Presque Isle, Lake Erie. About the Great Lakes. *Schoolcraft.* Astringent, antilithic. Big. Med. Bot. i. 66.

739. Gaultheria procumbens, *Linn.* Spicy wintergreen. May–Jul. *w.* Creeping evergreen. 4 to 6 i. ♃. Woods. rs. O. Ky. Stimulating, anodyne. Bart. i. 78. Big. ii. 27. Also astringent, emanagogue, antiseptic and diaphoretic. Beach's Mat. Med.

740. G. hispidula, *Muhl.* Creeping wintergreen. Ap.–May. *w.* Creeping, evergreen. ♄. Properties similar to the last?

741. Andromeda arborea, *Linn.* Sorrel tree. Jul. *w.* 40 f. ♄. Mar. O. Ky. Refrigerant, &c. Raf. Med. Fl. Leaves taste much like sorrel.

742. A. polifolia, *Michx.* Wild rosemary. June. *w* & *r.* 1 f. ♄. Sphagnous swamps. Mich. *Eat.*

743. A. calyculata, *Linn.* Ap.–May. *w.* 3 to 4 f. ♄. Swamps. Louisville. *M'Murt.*

744. A. mariana, *Linn.* June–Jul. *w* and pale *r.* 2 to 3 f. ♄. Sandy soils. Louisville. *M'Murt.*

745. A. racemosa, *Michx.* June–Jul. *w.* 4 to 6 f. ♄. Swamps and wet woods. Louisville. *M'Murt.*

§2. *Rhodoraceæ.*—Rose bay like.

746. Rhododendron maximum, *Linn.* American rose bay. June–Jul. Pale *r.* 10 to 15 f. ♄. Swamps. bg. Ky. *St.* Stimulant, narcotic, &c. Big. Med. Bot. iii. 101.
747. Kalmia latifolia, *Linn.* Mountain laurel. Calico bush. June–Jul. Rose coloured flowers. 4 to 10 f. ♄. Hills, &c. O. Ky. rs. Stimulant, narcotic, &c. Big. Med. Bot. i. 133.
748. K. glauca, *Ait.* Glaucous kalmia. June–Jul. Pale *r.* 12 to 18 i. ♄. Sphagnous swamps, Mich. *Eat.*
749. K. angustifolia, *Linn.* Sheep laurel. June–Jul. *r.* 12 to 18 i. ♄. Sandy woods. Louisville. *M'Murt.*
750. Azalea nudiflora, *Willd.* Early honeysuckle. May. *r.* 2 to 6 f. ♄. Ky. *St.*
751. A. viscosa, *Willd.* White honeysuckle. June. *w.* 5 f. ♄. Louisville. *M'Murt.*
752. Epigæa repens, *Linn.* Trailing arbutus. Ground laurel. *w* & *r.* Trailing evergreen. ♄. Pine hills. Newark, O. Ky.

CLXXII. *VACCINEÆ.*—The bilberry tribe.

The bark and leaves of these shrubs, are astringent, slightly tonic and stimulant. *Lind.*

753. Vaccinium stamineum, *Linn.* Deer berry. May–June. *w.* 2 to 3 f, ♄. Dry woods. rs. O. Ky. Used by some as a substitute for Arbutus uva-ursi. Mr. Miles, (page 86 of his Domestic Remedies,) says that this plant possesses diuretic, tonic and slightly astringent properties.
754. V. resinosum, *Ait.* Black whortleberry. May–June. Reddish *g.* 2 to 4 f. ♄. Woods and hills, Ky. *St.*
755. V. corymbosum, *Linn.* High wortleberry. June. Purplish *w.* 4 to 8 f. ♄. Swamps and wet woods, O. Ky. *St.*
756. V. frondosum, *Linn.* Whortleberry. Blue tangles. Ju. *w.* 3 to 5 f. ♄. Sandy woods, Ky. *St.*

757. Oxycoccus macrocarpus, *Pursh.* Cranberry. June. *w*, or pale *r*. Prostrate. ♄. bg. Near Lake Erie. The fruit possesses an agreeable tartness. Immense quantities are collected near the Lakes.

CLXXIII. *PYROLACEÆ.*—THE WINTERGREEN TRIBE.

758. Pyrola rotundifolia, *Linn.* Round-leaved wintergreen. Shin-leaf. July. *w*. 10 to 14 i. ♃. Woods. O.
759. P. elliptica, *Nutt.* July–Aug. *w*. 6 to 10 i. ♃. Dry woods, O.
760. P. minor, *Linn.* June. ♃. Mich. *Eat.*
761. Chimaphila umbellata, *Pursh. Eat.* Pipsissawa. Prince's pine. July. *g–w* and *p*. 4 to 6 i. ♃. Woods. Northern part of Ohio? Diuretic, tonic, vesicatory. Bart. i. 28. Big. ii. 15.
762. C. maculata, *Pursh, Linn.* Spotted wintergreen. July. *r–w*. 3 to 4 i. ♃. Sandy woods. Hills. O. Medicinal virtues similar to the preceding, though not so much concentrated.
763. Monotropa uniflora, *Linn.* Indian pipe. June. *w*. 5 to 8 i. ♃. sw. O. Ky.
764. M. lanuginosa, *Michx.* Tobacco-pipe. Aug. *w*. 4 to 6 i. ♃. Roots of trees. Ky. *St.* West Virginia. *Paddock.*

CLXXIV. *CAMPANULACEÆ.*—THE BELL-FLOWER TRIBE.

765. Campanula americana, *Linn.* Wheel-flower. July–Aug. *b*. 2 to 4 f. ♃. Thickets. O. Ky.
766. C. amplexicaulis, *Michx.* Clasping bell-flower. May–July. *p*. 8 to 12 i. ⊙. Fields. W.
767. C. aparinoides, *Pursh.* Prickly bell-flower. June–July. *w*. 1 f. ⊙. wp. O.

CLXXV. *LOBELIACEÆ.*—THE LOBELIA TRIBE.

768. Lobelia cardinalis, *Linn.* Cardinal flower. July–Aug. *r*. 2 to 3 f. ♃. Wet woods. O. Ky. Acrid, anthelmintic. Bart. ii. 180.
769. L. syphilitica, *Linn.* Blue cardinal flower. Sept. *b* & *w*. 2 to 3 f. ♃. wp. ow. bg. O. Ky. Diuretic, purgative, anti-syphilitic. Bart. ii. 211.
770. L. puberula, *Michx.* Sept. *b*. 2 to 3 f. ♃. mh. bg. O. Ky.
771. L. inflata, *Linn.* Indian tobacco. Aug. Pale *b*. 1 f. ⊙. Fields and woods. W. Emetic, sudorific, expectorant. Bart. i. 189. Big. i. 177. Beach's Mat. Med.

772. L. claytoniana, *Michx.* Clayton's lobelia. Jul.–Aug. Pale *b.* 1½ to 2 f. ♃. dp. wp. O. Ky.
773. L. kalmii, *Linn.* Kalm's lobelia. Jul.–Aug. *b.* 12 to 20 i. ♃. wp. O.

CLXXXI. *CUCURBITACEÆ.*—The gourd tribe.

774. Momordica echinata, *Muhl.* Balsam apple. Aug. *w.* Climbing. ⊙. Banks of streams. O. Mich. Mo. Riv.
775. Sicyos angulatus, *Linn.* Single-seeded cucumber. June–Jul. *w–g.* Procument vine. ⊙. bt. Banks of streams, W.

CLXXXII. *PLANTAGINEÆ.*—The rib-grass tribe.

776. Plantago cordata, *Lam.* P. asiatica, *Spreng?* June–Jul. 12 to 18 i. ♃. rb. Banks of streams. W.
777. P. media, *Linn.* Jul. 3 to 8 i. ♃. Fields. Miami country.
778. P. major, *Linn.* Common plantain. Aug. 8 to 12 i. ♃. Fields. Intr. W. Healing, refrigerant, antiseptic. *Beach.* Other species have similar virtues.
779. P. glabra, *Nutt.* Arid situations. Plt. *Jas.* Germantown, O. Frank. Mar. Wor. O?
780. P. lanceolata, *Linn.* Rib-wort. Snake plantain. May–Sept. 6 to 12 i. ♃. Pastures, &c. Intr. Ky. *St.* Opposite Cin. O.
781. P. virginica, *Linn.* Dwarf plantain. June. 3 to 8 i. ♂. Sandy soils. Ky. *St.*
782. P. pusilla, *Nutt.* June. 2 to 3 i. ⊙. Sandy hills. W. *Bk.* Mo. Ter. *Nutt.*
783. P. gnaphalioides, *Nutt.* Jul. ⊙. Mo. Ter. *Nutt.*

CLXXXIV. *DIPSACEÆ.*—The scabious tribe.

784. Dipsacus sylvestris, *Linn,* Wild teasel. Jul. *p.* or *b.* 3 to 5 f. ♂. Fields. bt. W.

CLXXXV. *VALERIANEÆ*—The valerian tribe.

785. Valeriana pauciflora, *Nutt.* i. 20. Western valerian. *r.* 1½ to 3 f. ♃. sw. bt. W. Tonic, antispasmodic, vermifugal(?)
786. Valerianella radiata, *De Cand.* Wild lamb lettuce. May. *w.* 1 f. ⊙. Fields, W.
787. V. chenopodifolia, *Pursh, De Cand.* 8 i. ⊙. Open woods and meadows, Miami country. *Frank.*

Note.—A supplementary table will be given, at the conclusion of this Synopsis, in the next number of the Western Journal, which will make the reference to authorities for specific names complete.

